# Sequential Infection with Influenza A Virus Followed by Severe Acute Respiratory Syndrome Coronavirus 2 (SARS-CoV-2) Leads to More Severe Disease and Encephalitis in a Mouse Model of COVID-19

**DOI:** 10.3390/v16060863

**Published:** 2024-05-28

**Authors:** Jordan J. Clark, Rebekah Penrice-Randal, Parul Sharma, Xiaofeng Dong, Shaun H. Pennington, Amy E. Marriott, Stefano Colombo, Andrew Davidson, Maia Kavanagh Williamson, David A. Matthews, Lance Turtle, Tessa Prince, Grant L. Hughes, Edward I. Patterson, Ghada Shawli, Daniele F. Mega, Krishanthi Subramaniam, Jo Sharp, Joseph D. Turner, Giancarlo A. Biagini, Andrew Owen, Anja Kipar, Julian A. Hiscox, James P. Stewart

**Affiliations:** 1 Department of Infection Biology & Microbiomes, Institute of Infection, Veterinary and Ecological Sciences, University of Liverpool, Liverpool L3 5RF, UKr.penrice-randal@liverpool.ac.uk (R.P.-R.); parul13@liverpool.ac.uk (P.S.); tprince@liverpool.ac.uk (T.P.); g.shawli@liverpool.ac.uk (G.S.); anja.kipar@uzh.ch (A.K.); 2 Department of Tropical Disease Biology, Centre for Drugs and Diagnostics, Liverpool School of Tropical Medicine, Liverpool L3 5QA, UKjoseph.turner@liverpool.ac.uk (J.D.T.);; 3 School of Cellular and Molecular Medicine, Faculty of Life Sciences, University of Bristol, Bristol BS8 1QU, UK; andrew.davidson@bristol.ac.uk (A.D.); d.a.matthews@bristol.ac.uk (D.A.M.); 4 Department of Clinical Infection Microbiology and Immunology and NIHR Health Protection Research Unit for Emerging and Zoonotic Infections, Institute of Infection, Veterinary and Ecological Sciences, University of Liverpool, Liverpool L69 3BX, UK; 5 Tropical & Infectious Disease Unit, Royal Liverpool University Hospital, Liverpool L7 8YE, UK; 6 Departments of Vector Biology and Tropical Disease Biology, Centre for Neglected Tropical Disease, Liverpool School of Tropical Medicine, Liverpool L3 5QA, UK; grant.hughes@lstmed.ac.uk (G.L.H.);; 7 Department of Pharmacology and Therapeutics, Centre of Excellence in Long-acting Therapeutics (CELT), University of Liverpool, Liverpool L69 3BX, UK; momeejl2@liverpool.ac.uk (J.S.); aowen@liverpool.ac.uk (A.O.); 8 Laboratory for Animal Model Pathology, Institute of Veterinary Pathology, Vetsuisse Faculty, University of Zurich, 8057 Zürich, Switzerland; 9 College of Veterinary Medicine, Northwest A&F University, Yangling, Xianyang 712100, China; 10 Infectious Diseases Horizontal Technology Centre (ID HTC), Agency for Science, Technology and Research (A*STAR), Singapore 138632, Singapore; 11 Department of Infectious Disease, University of Georgia, Athens, GA 30602, USA

**Keywords:** Severe Acute Respiratory Syndrome Coronavirus-2, influenza A virus, co-infection, transcriptomic signatures

## Abstract

COVID-19 is a spectrum of clinical symptoms in humans caused by infection with SARS-CoV-2. The coalescence of SARS-CoV-2 with seasonal respiratory viruses, particularly influenza viruses, is a global health concern. To understand this, transgenic mice expressing the human ACE2 receptor (K18-hACE2) were infected with influenza A virus (IAV) followed by SARS-CoV-2 and the host response and effect on virus biology was compared to K18-hACE2 mice infected with IAV or SARS-CoV-2 alone. The sequentially infected mice showed reduced SARS-CoV-2 RNA synthesis, yet exhibited more rapid weight loss, more severe lung damage and a prolongation of the innate response compared to the singly infected or control mice. Sequential infection also exacerbated the extrapulmonary encephalitic manifestations associated with SARS-CoV-2 infection. Conversely, prior infection with a commercially available, multivalent live-attenuated influenza vaccine (Fluenz Tetra) elicited the same reduction in SARS-CoV-2 RNA synthesis, albeit without the associated increase in disease severity. This suggests that the innate immune response stimulated by IAV inhibits SARS-CoV-2. Interestingly, infection with an attenuated, apathogenic influenza vaccine does not result in an aberrant immune response and enhanced disease severity. Taken together, the data suggest coinfection (‘twinfection’) is deleterious and mitigation steps should be instituted as part of the comprehensive public health and management strategy of COVID-19.

## 1. Introduction

Coronaviruses were once described as the backwater of virology, but the last two decades have seen the emergence of three major coronavirus threats [1]. First, severe acute respiratory syndrome coronavirus (SARS-CoV) in China in 2003. Second, Middle East respiratory syndrome coronavirus (MERS-CoV) [2] in Saudi Arabia in 2012 and most recently, SARS-CoV-2 originating in China in 2019. Whilst SARS-CoV was eradicated, both MERS-CoV and SARS-CoV-2 represent ongoing health threats and a greater understanding is required to develop robust interventions for future emergent coronaviruses. Coronaviruses share similar genome architectures and disease profiles and generally cause respiratory and gastrointestinal illnesses [1]. However, some animal/avian coronaviruses can also affect other organ systems, causing, for example, demyelination and nephritis. The sheer scale of the coronavirus disease 2019 (COVID-19) outbreak has highlighted hitherto unexpected aspects of coronavirus infection in humans, including long term disease complications once the virus has been cleared.

The infection of humans with SARS-CoV-2 results in a range of clinical courses, from asymptomatic to severe infection and subsequent death in not only at-risk individuals, but also a small proportion of otherwise healthy individuals across all age groups. Severe infection in humans is typified by cytokine storms [3,4], pneumonia and kidney failure. The examination of post-mortem tissue reveals a disconnect between viral replication and immune pathology [5]. A range of other clinical signs also occur, including gastrointestinal symptoms such as vomiting, diarrhoea, abdominal pain and loss of appetite as well as a loss of taste and smell (anosmia). A small number of patients have no overt respiratory symptoms at all. 

Respiratory infections in humans and animals can also be synergistic, in which an initial infection can exacerbate a secondary infection or vice versa. When multiple pathogens are in circulation at the same time, this can lead to cooperative or competitive forms of pathogen–pathogen interactions [6]. This was evident during the 1918 Spanish influenza A virus (IAV) outbreak where secondary bacterial pneumonia was thought to be a leading cause of death [7]. Coinfections in other viral diseases, such as in patients with Ebola virus disease, have also been shown to contribute to the host response and outcome [8]. Global influenza cases decreased due to the lockdowns implemented to contain the SARS-CoV-2 spread [9,10,11]; the lifting of these lockdowns in 2021 and 2022 has resulted in the return of seasonal influenza outbreaks (Weekly U.S. Influenza Surveillance Report, CDC, https://www.cdc.gov/flu/weekly/index.htm; accessed on 1 March 2024). With ongoing SARS-CoV-2 waves caused by emerging variants of concern (VOCs) and returning seasonal IAV outbreaks, coinfection with these respiratory pathogens is likely and this may exacerbate the clinical disease and, potentially, the outcome.

Previous work has shown that coinfections are present in patients with severe coronavirus infection. For SARS-CoV, the co-circulation of human metapneumovirus was reported in an outbreak in Hong Kong. However, the data suggested that outcomes were not different between patients with identified coinfections and those with SARS-CoV infection alone [12]. For MERS-CoV, four cases of coinfection with IAV were described and although no data were presented on the severity of symptoms, this sample size would have been too small to allow any meaningful conclusions [13]. Post-mortem studies from patients with COVID-19 in Beijing (n = 85) identified IAV in 10% of patients, influenza B virus in 5% and respiratory syncytial virus (RSV) in 3% of patients, but the absence of a carefully selected control arm prohibits conclusions to be drawn [14]. Additionally, there have been several case reports of coinfections with IAV and SARS-CoV-2 in humans with severe outcomes [15,16,17,18,19,20], with one study from the UK reporting that patients with a coinfection exhibited a ~6 times higher risk of death [21]. Whilst this suggests that coinfection is synergistic, this study also found that the risk of testing positive for SARS-CoV-2 was 68% lower among individuals who were positive for IAV infection, implying that the two viruses may competitively exclude each other [21].

Whilst the analysis of post-mortem tissue is extremely informative in what may have led to severe coronavirus infection and death, the analysis of the disease in severe (but living) cases is naturally restricted by what samples can be collected (e.g., blood, nasopharyngeal swabs and bronchioalveolar lavages). Therefore, animal models of COVID-19 present critical tools to fill knowledge gaps for the disease in humans and for screening therapeutic or prophylactic interventions. Compatibility with a more extensive longitudinal tissue sampling strategy and a controlled nature of infection are key advantages of animal models [22]. Studies in an experimental mouse model of SARS-CoV showed that the coinfection of a respiratory bacterium exacerbated pneumonia [23]. Different animal species can be infected with wild-type SARS-CoV-2 to serve as models of COVID-19 and these include mice, hamsters, ferrets, rhesus macaques and cynomolgus macaques. The K18-hACE2 transgenic (K18-hACE2) mouse, where hACE2 expression is driven by the epithelial cell cytokeratin-18 (K18) promoter, was developed to study SARS-CoV pathogenesis [24]. This mouse has been used extensively as a model that mirrors many features of severe COVID-19 in humans to develop understanding of the mechanistic basis of lung disease and to test pharmacological interventions [25,26].

IAV and SARS-CoV-2 co- and sequential infections have already been investigated, with partly controversial results. In some studies, coinfection led to more severe pneumonia [27,28,29,30]. A similar study in hamsters found reduced disease with IAV followed by SARS-CoV-2 infection [31]. Another study then found evidence that co-infection with SARS-CoV-2 leads to more widespread IAV infection in the lungs [32], while a third study described impaired IAV replication in lungs when hamsters were infected early and 10 days after SARS-CoV-2 inoculation, but no effect was noted when IAV infection took place on day 21 post-SARS-CoV-2 infection [33].

With the likelihood of flu seasons concomitant with waves of SARS-CoV-2 infections, there is an obvious public health concern about the possibility of enhanced morbidity and mortality in coinfected individuals. The aim of this work is to use an established pre-clinical model of COVID-19 to study the consequences of coinfection with SARS-CoV-2 and IAV, defining the associated clinical, pathological and transcriptomic signatures.

## 2. Materials and Methods

### 2.1. Cell Culture and Viruses

Influenza virus A/HKx31 (X31, H3N2) was propagated in the allantoic cavity of 9-day-old embryonated chicken eggs at 35 °C for 72 h. Titres were determined by an influenza plaque assay using MDCK cells.

Vero E6 cells (C1008; African green monkey kidney cells) were obtained from Public Health England and maintained in Dulbecco’s minimal essential medium (DMEM) containing 10% foetal bovine serum (FBS) and 0.05 mg/mL gentamycin at 37 °C with 5% CO_2_.

A UK strain of SARS-CoV-2 (hCoV-2/human/Liverpool/REMRQ0001/2020), which was cultured from a nasopharyngeal swab from a patient, was used for these studies [34]. Virus was cultured at an MOI of 0.001 in Vero E6 cells with DMEM containing 4% FBS and 0.05 mg/mL gentamycin at 37 °C with 5% CO_2_ and was harvested 48 h post-inoculation. Virus stocks were stored at −80 °C. The intracellular viral genome sequence and the titre of virus in the supernatant were determined. Direct RNA sequencing was performed as described previously [35] and an inhouse script was used to check for deletions in the mapped reads. The Illumina reads were mapped to the England/2/2020 genome using HISAT and the consensus genome was called using an in-house script based on the dominant nucleotide at each location on the genome. 

Fluenz Tetra (AstraZeneca) is a commercially available quadrivalent live vaccine composed of attenuated A/Victoria/2570/2019 (H1N1) pdm09, A/Cambodia/e0826360/2020 (H3N2), B/Washington/02/2019 and B/Phuket/3073/2013.

### 2.2. Biosafety

Biosafety. All work was performed in accordance with risk assessments and standard operating procedures approved by the University of Liverpool Biohazards Sub-Committee and by the UK Health and Safety Executive. Work with SARS-CoV-2 was performed at containment level 3 by personnel equipped with respirator airstream units with filtered air supply.

### 2.3. Mice

Animal work was approved by the local University of Liverpool Animal Welfare and Ethical Review Body and performed under UK Home Office Project Licence PP4715265. Female mice aged 6–8 weeks carrying the human ACE2 gene under the control of the keratin 18 promoter (K18-hACE2; formally B6.Cg-Tg(K18-ACE2)2Prlmn/J) were purchased from Jackson Laboratories. Mice were maintained under SPF barrier conditions in individually ventilated cages.

### 2.4. Virus Infection

**Figure 1:**
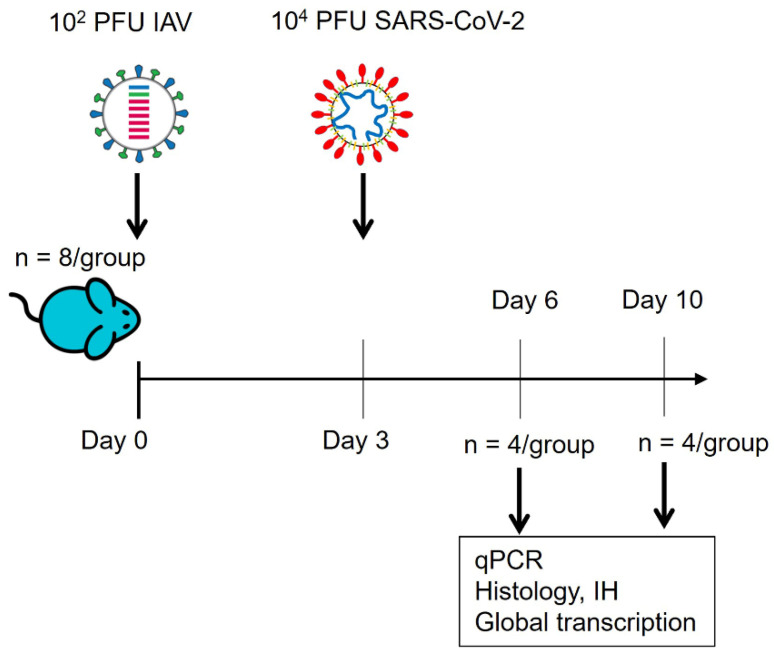
Schematic diagram of the experimental design for infection of K18-hACE2 mice sequentially with IAV strain A/X31 and SARS-CoV-2 (hCoV-19/England/Liverpool_REMRQ0001/2020). qPCR: quantitative RT-PCR for viral load; IH: immunohistology for SARS-CoV-2 nucleoprotein and IAV antigen.

Animals were randomly assigned into multiple cohorts. For IAV infection, mice were anaesthetised lightly with KETASET i.m. and inoculated intra-nasally with 10^2^ PFU IAV X31 in 50 μL sterile PBS (n = 16) or mock-infected with 50 μL sterile PBS (n = 12). Three days post-IAV infection, eight mice from the IAV-infected group and eight mice from the mock-infected group were anaesthetised lightly with isoflurane and inoculated intra-nasally with 50 μL containing 10^4^ PFU SARS-CoV-2 in PBS. The remaining eight mice infected with IAV were mock-infected with 50 μL sterile PBS. For Fluenz Tetra immunisation, mice (n = 16) were anaesthetised with isoflurane and intranasally inoculated with 50 μL of vaccine formulation. Each 50 μL of Fluenza Tetra contains around 2 × 10^6±4^ of A/Victoria/2570/2019 (H1N1)pdm09, A/Cambodia/e0826360/2020 (H3N2), B/Washington/02/2019 and B/Phuket/3073/2013. On day 3 post-inoculation, eight mice were anaesthetised and infected with 10^4^ PFU SARS-CoV-2 in PBS and eight were mock-infected with 50 μL sterile PBS. Therefore, six experimental groups (n = 4) were generated: control, IAV only, SARS-CoV-2 only, Fluenz Tetra only as well as IAV + SARS-CoV-2 and Fluenz Tetra and SARS-CoV-2 (Figure 1, Table 1).

Mice were sacrificed 6 or 10 days after the first infection (Figure 1) by an overdose of pentabarbitone and immediately dissected after death. Tissue samples (lungs and brains) were collected for downstream processing.

### 2.5. Histology and Immunohistology

From all animals the right lung and brain were collected and fixed in 10% neutral buffered formal saline for 24–48 h, then transferred to 70% ethanol until they were trimmed (longitudinal section of the lung, coronal section of the brain) and routinely paraffin-wax embedded. Consecutive sections (3–5 μm) were either stained with hematoxylin and eosin (HE) or used for immunohistology (IH). IH was performed to detect viral antigens and to identify macrophages, T cells and B cells using the horseradish peroxidase (HRP) method. The following primary antibodies were applied: goat anti-IAV (H1N1 virions; Meridian Life Sciences Inc., Memphis, TN, USA: B65141G), rabbit anti-SARS-CoV nucleocapsid protein (Rockland Immunochemicals Inc., Limerick, ME, USA; 200-402-A50), rabbit anti-Iba-1 (antigen: AIF1; Wako Chemicals, Osaka, Japan; macrophage marker), monoclonal rabbit anti-mouse CD3 (clone SP7: Spring Bioscience, Ventana Medical Systems, Tucson, AZ, USA; T cell marker) and rat anti-mouse CD45R (clone B220, BD Biosciences; B cell marker), following previously published protocols [36]. Briefly, after deparaffination, sections underwent antigen retrieval in citrate buffer (pH 6.0; Agilent, Glastrup, Denmark) or Tris/EDTA buffer (pH 9) for 20 min at 98 °C, followed by incubation with the primary antibodies (diluted in dilution buffer, Agilent) overnight at 4 °C for SARS-CoV-2 and for 1 h at RT for all other antigens. This was followed by blocking of endogenous peroxidase (peroxidase block, Agilent) for 10 min at RT and incubation with the secondary antibodies, EnVision+/HRP Rabbit (Agilent) for Iba1, CD3 and SARS-CoV, EnVision+/HRP Rat (Agilent) for CD45R and rabbit anti-goat Ig/HRP (Agilent) for IAV, for 30 min at RT and EnVision FLEX DAB+ Chromogen in Substrate buffer (Agilent) for 10 min at RT, all in an autostainer (Agilent Dako, Glastrup, Denmark). Sections were subsequently counterstained with hematoxylin.

Lungs and brain from two wild-type C57BL6/J mice infected intranasally with 10^2^ PFU IAV X31 in 50 μL sterile PBS and sacrificed at 6 days post-infection served to assess any effect of hACE2 expression in the course of IAV infection. A formalin-fixed, paraffin-embedded cell pellet infected with IAV for 24 h served as positive control for the IAV staining and the spleen of a control mouse as positive control for the leukocyte markers. Sections incubated without the primary antibodies served as negative controls.

### 2.6. RNA Extraction and DNase Treatment 

The upper lobes of the right lung were dissected and homogenised in 1 mL of TRIzol reagent (Thermo Fisher Scientific, Waltham, MA, USA) using a Bead Ruptor 24 (Omni International, Kennesaw, GA, USA) at 2 m per second for 30 s. The homogenates were clarified by centrifugation at 12,000× *g* for 5 min before full RNA extraction was carried out according to manufacturer’s instructions. RNA was quantified and quality assessed using a Nanodrop (Thermo Fisher Scientific) before a total of 1 μg was DNase treated using the TURBO DNA-free™ Kit (Thermo Fisher Scientific) as per manufacturer’s instructions.

### 2.7. Quantitative (q)RT-PCR for Viral Load

Viral loads were quantified using the GoTaq^®^ Probe 1-Step RT-qPCR System (Promega). For quantification of SARS-CoV-2, the nCOV_N1 primer/probe mix from the SARS-CoV-2 (2019-nCoV) CDC qPCR Probe Assay (IDT) were utilised while the standard curve was generated via 10-fold serial dilution of the 2019-nCoV_N_Positive Control (IDT) from 10^6^ to 0.1 copies/reaction. The E sgRNA primers and probe have been previously described [37] and were utilised at 400 nM and 200 nM, respectively. Murine 18S primers and probe sequences were utilised at 400 nM and 200 nM, respectively. The IAV primers and probe sequences are published as part of the CDC IAV detection kit (20403211). The IAV reverse genetics plasmid encoding the NS segment was diluted 10-fold from 10^6^ to 0.1 copies/reaction to serve as a standard curve. The thermal cycling conditions for all qRT-PCR reactions were as follows: 1 cycle of 45 °C for 15 min and 1 cycle of 95 °C followed by 40 cycles of 95 °C for 15 s and 60 °C for 1 min. The 18s standard was generated by the amplification of a fragment of the murine 18S cDNA using the primers F: ACCTGGTTGATCCTGCCAGGTAGC and R: AGC CAT TCG CAG TTT TGT AC. Similarly, the E sgRNA standard was generated by PCR using the qPCR primers. cDNA was generated using the SuperScript IV reverse transcriptase kit (Thermofisher) and PCR carried out using Q5^®^ High-Fidelity 2· Master Mix (New England Biolabs, Ipswich, MA, USA) as per manufacturer’s instructions. Both PCR products were purified using the QIAquick PCR Purification Kit (Qiagen, Hilden, Germany) and serially diluted 10-fold from 10^10^ to 10^4^ copies/reaction to form the standard curve.

### 2.8. Illumina RNA Sequencing

Following the manufacturers’ protocols, total RNA from lung tissue were used as input material in to the QIAseq FastSelect–rRNA HMR (Qiagen) protocol to remove cytoplasmic and mitochondrial rRNA with a fragmentation time of 7 or 15 min. Subsequently, the NEBNext^®^ Ultra™ II Directional RNA Library Prep Kit for Illumina^®^ (New England Biolabs) was used to generate the RNA libraries, followed by 11 cycles of amplification and purification using AMPure XP beads. Each library was quantified using Qubit and the size distribution assessed using the Agilent 2100 Bioanalyser and the final libraries were pooled in equimolar ratios. The raw fastq files (2 × 150 bp) generated by an Illumina^®^ NovaSeq 6000 (Illumina^®^, San Diego, CA, USA) were trimmed to remove Illumina adapter sequences using Cutadapt v1.2. [38]. The option “−O 3” was set, so that the 3′ end of any readings which matched the adapter sequence, being greater than 3 bp, was trimmed off. The readings were further trimmed to remove low-quality bases, using Sickle v1.200 (https://github.com/najoshi/sickle (accessed on 1 March 2024)) with a minimum window quality score of 20. After trimming, readings shorter than 10 bp were removed.

### 2.9. RNA Sequencing Bioinformatic Analysis

Trimmed paired-end sequencing readings were inputted into salmon (v1.5.2) using the -l A –validateMappings –SeqBias –gcBias parameters. Quant files generated with salmon were imported into RStudio (4.1.1) using tximport to infer gene expression [39]. The edgeR package (3.34.1) was used to normalise sequencing libraries and identify differentially expressed genes, defined as at least a 2-fold difference from the mock-infected group and a false discovery rate (FDR) less than 0.05 51. Principle component Analysis (PCA), volcano plots, heatmaps and Venn diagrams were produced in R studio using the following packages: edgeR, ggplot2 (3.3.5) and pheatmap (1.0.12). Differential gene expression data were used for gene ontology enrichment analysis of biological process terms in each group using the compareCluster function with enrichGO in the ClusterProfiler package (4.0.5) programme in R [39]. Code used to analyse data is available at https://github.com/Hiscox-lab/k18-hACE2-coinfection-transcriptomics (accessed on 1 March 2024). Sequencing readings are available under BioProject ID: PRJNA914664 on Short Read Archive (SRA).

### 2.10. Statististical Analysis

Data were analysed using the Prism package (version 5.04 Graphpad Software). *p* values were set at 95% confidence interval. A repeated-measures two-way ANOVA (Bonferroni post-test) was used for time-courses of weight loss; two-way ANOVA (Bonferroni post-test) was used for other time-courses; log-rank (Mantel–Cox) test was used for survival curves. All differences not specifically stated to be significant were not significant (*p* > 0.05). For all figures, * *p* < 0.05, ** *p* < 0.01 and *** *p* < 0.001.

## 3. Results

### 3.1. Sequential Infection with Pathogenic IAV, but Not Fluenz Tetra Plus SARS-CoV-2, Leads to Enhanced Disease

To assess the impact of a sequential infection with influenza virus followed by SARS-CoV-2 infection, the established K18-hACE2 mouse model of SARS-CoV-2 was utilised [24]. We used a clinical isolate of SARS-CoV-2 (strain hCoV-19/England/Liverpool_REMRQ0001/2020) [34] and the non-lethal IAV strain A/X31. Importantly, sequence of the virus stock demonstrated that this isolate did not contain deletions or mutations of the furin cleavage site in the S protein [35]. 

**Figure 2:**
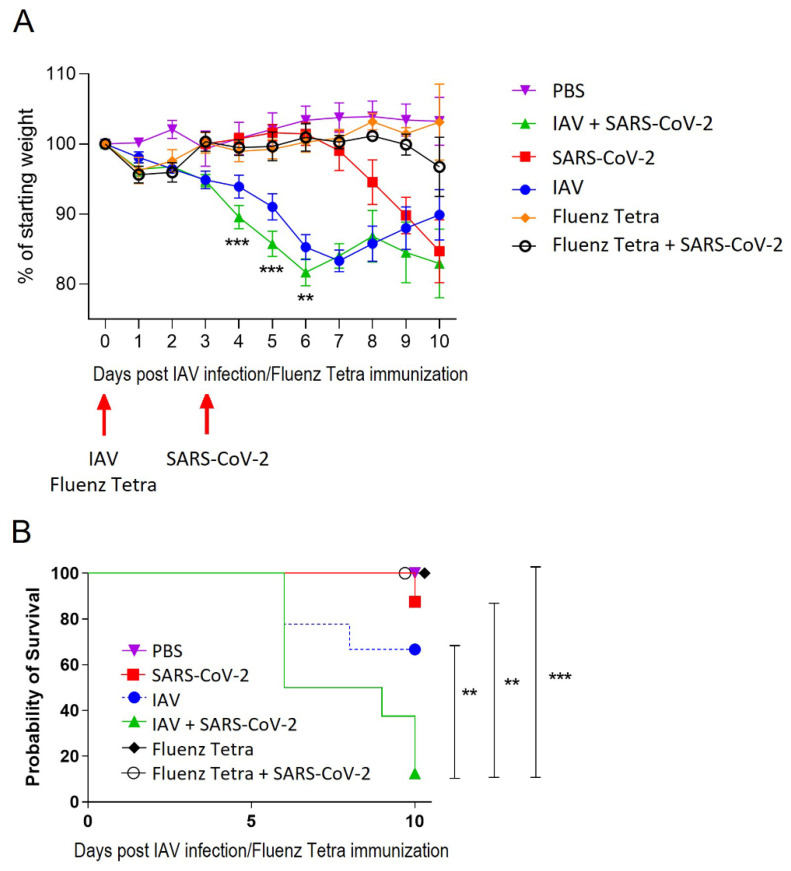
Coinfection with IAV and SARS-CoV-2 leads to enhanced weight loss and more rapid mortality. K18-hACE2 mice were challenged intranasally with IAV strain X31 (10^2^ PFU) or immunised with Fluenz Tetra and challenged 3 days later with 10^4^ PFU SARS-CoV-2. (**A**) Mice were monitored for weight loss at indicated time points (n = 8 until day 3 post-infection, then n = 4). Data represent the mean value ± SEM. Comparisons were made using a repeated-measures two-way ANOVA (Bonferroni post-test). (**B**) Survival was assessed at indicated time points (n = 8). Comparisons were made using log-rank (Mantel–Cox) test. ** represents *p* < 0.01; *** represents *p* < 0.001.

Control mice maintained their body weight throughout. Mice infected with IAV displayed a typical pattern of weight loss, reaching a nadir (mean 17% loss) at 7 dpi before starting recovery. SARS-CoV-2-infected animals started to lose weight at day 7 (4 dpi) and carried on losing weight up to day 10 (mean 15% loss). Mice infected with IAV then SARS-CoV-2 had significantly accelerated weight loss as compared with IAV-infected mice from day 4; this was most severe at day 6 (mean 19%), followed by recovery to day 8 (mean 14% loss) before losing weight again (mean 17% loss) (Figure 2A). As well as accelerated weight loss, IAV + SARS-CoV-2-infected mice exhibited more severe respiratory signs and a significantly more rapid mortality (assessed by a humane endpoint of 20% weight loss) as compared with mice infected with either virus alone (Figure 2B). 

To investigate the effect of infection with a non-pathogenic vaccine formulation of influenza, K18-hACE2 mice were dosed intranasally with Fluenz Tetra 3 days prior to SARS-CoV-2 infection (Table 1). Mice that received Fluenz Tetra only showed no significant deviation in weight loss in comparison to the PBS controls (Figure 2A). Mice that received Fluenz Tetra immunisation followed by a SARS-CoV-2 infection initially had a slight reduction in weight at day 1 and day 2; however, between day 3 and day 8, they resembled the PBS control mice, although at day 10, two coinfected mice had lost 13% and 9% of their body weight while two continued to gain weight (Figure 2A). The animals showing weight loss did not exhibit respiratory signs associated with SARS-CoV-2 infection or coinfection.

### 3.2. Coinfection of SARS-CoV-2 and IAV Results in Reduced SARS-CoV-2 Viral Load at Day 6 but Not Day 10 Post-IAV Infection

**Figure 3:**
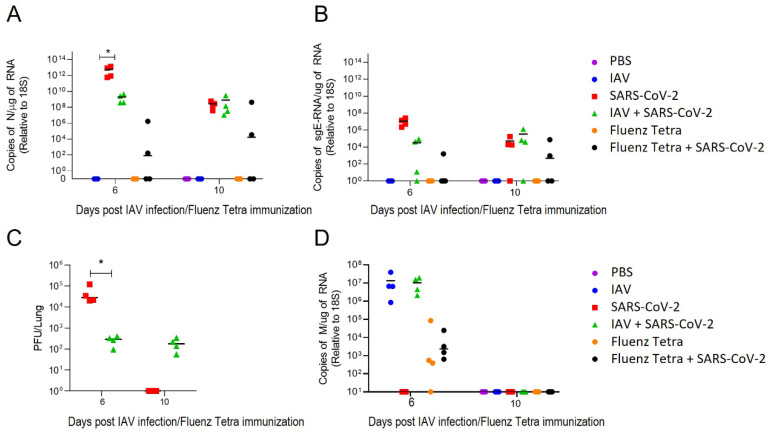
Viral loads and SARS-CoV-2 sgRNA levels in singly and coinfected mice. K18-hACE2 mice were challenged intranasally with IAV strain X31 (10^2^ PFU) and 3 days later with 10^4^ PFU SARS-CoV-2 (n = 4). RNA extracted from lungs was analysed for virus levels by qRT-PCR. Assays were normalised relative to levels of 18S RNA. Data for individual animals are shown with the median value represented by a black line. (**A**) SARS-CoV-2 viral load was determined using qRT-PCR for the N gene. (**B**) Levels of SARS-CoV-2 sub-genomic RNA (sgRNA) for the E gene. (**C**) SARS-CoV-2 titre was determined by plaque assay on Vero E6 cells. (**D**) IAV load was determined using RT-PCR for the M gene. Comparisons were made using two-way ANOVA (Bonferroni post-test). * Represents *p* < 0.05.

In order to determine whether the sequential infection of IAV and SARS-CoV-2 was cooperative or competitive, the total RNA was extracted from the lungs of the K18-hACE2 mice and viral loads were quantified using qRT-PCR. At day 6 (3 dpi), the SARS-CoV-2-infected mice exhibited 10,000-fold higher levels of viral load than at day 10 (7 dpi) (mean 6 × 10^12^ vs. 2.8 × 10^8^ copies of N/μg of RNA), indicating peak viral replication before the onset of clinical signs at 4 dpi (Figure 3A). At this time point, the mice infected with SARS-CoV-2 alone displayed significantly higher levels of viral RNA than the mice with IAV and SARS-CoV-2 coinfection (mean 6 × 10^12^ vs. ~2 × 10^9^ copies of N/μg of RNA) (Figure 3A). The mice preimmunised with Fluenz Tetra exhibited reduced levels of viral RNA compared to both SARS-CoV-2 singly and IAV-coinfected mice, with 2/4 mice exhibiting no detectable viral RNA. However, by day 10, the SARS-CoV-2 and IAV X31-coinfected and singly infected mice exhibited nearly identical levels of SARS-CoV-2 RNA (mean 2 × 10^8^ vs. 8.1 × 10^8^ copies of N/μg of RNA) (Figure 3A). Conversely, SARS-CoV-2 RNA levels were undetectable in 2/4 of the mice pre-immunised with Fluenz Tetra, with only one animal displaying similar levels of viral RNA to the SARS-CoV-2 singly and IAV-coinfected animals. The levels of the infectious virus generally corresponded with the copies of N RNA, except at day 10, when there was no infectious virus in mice infected with SARS-CoV-2 alone, whereas the level of infectious virus in coinfected mice was similar at both day 6 and day 10 (10^2^ PFU/lung) (Figure 3C). Conversely, at day 6, the mice infected with IAV alone showed similar levels of IAV RNA compared to the coinfected mice (mean 1.3 × 10^7^ vs. 1 × 10^7^ copies of M/μg of RNA) and by day 10, both the singly infected mice and coinfected mice did not display any detectable IAV RNA, demonstrating similar levels of IAV clearance (Figure 3D). Fluenz Tetra-immunised mice displayed reduced levels of IAV viral RNA compared to IAV-infected mice (mean 5 × 10^3^), in line with the reduced replication expected of attenuated IAV. To investigate viral replication, qPCR was employed to quantify viral subgenomic mRNA (sgRNA) transcripts. The amount of sgRNA in the SARS-CoV-2-infected mice was concomitant with the viral load, appearing to be 100-fold higher at day 6 (3 dpi) than day 10 (7 dpi) (mean 6.2 × 10^6^ vs. 5.4 × 10^4^ copies of E sgRNA/μg of RNA) (Figure 3B). Similarly, the amount of sgRNA was significantly lower in the coinfected mice compared to the SARS-CoV-2 singly infected mice (mean 6.2 × 10^6^ vs. 1.7 × 10^4^ copies of E sgRNA/μg of RNA); however, by day 10 (7 dpi), both coinfected and singly infected mice displayed similar levels of sgRNA (mean 5.4 × 10^4^ vs. 3.5 × 10^5^ copies of E sgRNA/μg of RNA) (Figure 3B). Fluenz Tetra-immunised mice exhibited further reduced levels of sgRNA at day 6, with detectable sgRNA in one mouse only; however, by day 10, 2/4 mice displayed detectable sgRNA. 

### 3.3. Coinfection Leads to Complementary and Enhanced Pathological Processes

Transgenic mice carrying the human ACE2 receptor under the control of the keratin 18 promoter (K18-hACE2), a widely used COVID-19 model, also served in the current co-infection study. 

**Figure 4:**
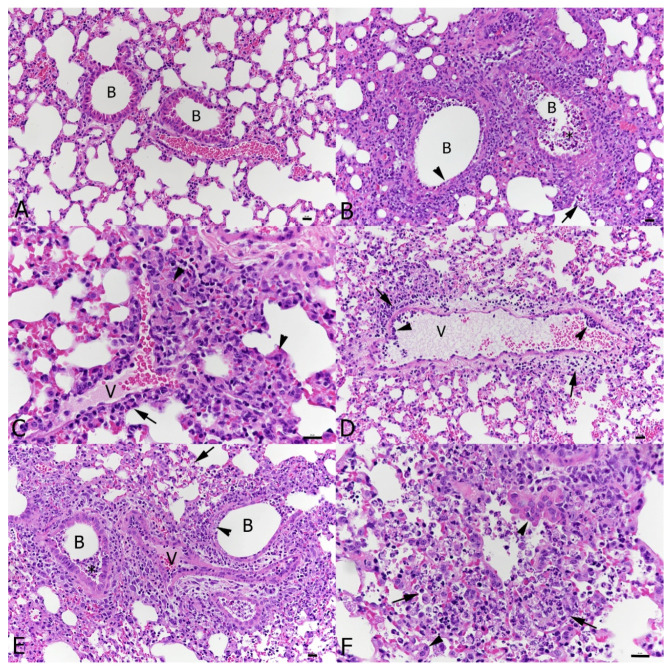
Lungs, K18-hACE2 transgenic mice, after mock infection or at day 6 post-infection with IAV and day 3 post-infection with SARS-CoV-2 in single and double infections. (**A**) Mock-infected control animal. Normal lung. (**B**) IAV-infected animal; 6 dpi. Bronchioles (**B**) exhibit necrosis (arrowhead) of a variable number of epithelial cells and contain degenerate cells in the lumen (*). The parenchyma adjacent to affected bronchioles often exhibits individual alveoli with necrotic epithelial cells (arrow). (**C**,**D**) SARS-CoV-2-infected animal; 3 dpi. The parenchyma exhibits multifocal activation of type II pneumocytes ((**C**): arrowheads) and leukocyte recruitment, represented by leukocyte emigration from vessels (V) ((**D**): arrowheads) and perivascular infiltrates (arrows). (**E**,**F**) IAV (6 dpi) and SARS-CoV-2 (3 dpi) double infection. The IAV associated changes, with necrosis of bronchiolar epithelial cells ((**E**): arrowhead), debris in bronchiolar lumina (*), focal necrosis of alveolar epithelial cells (arrows) as well some activation and hyperplasia of type II pneumocytes ((**F**): arrowheads), dominate the histological picture. B: bronchiole; V: vessel. HE stain; Bars = 20 μm.

**Figure 5:**
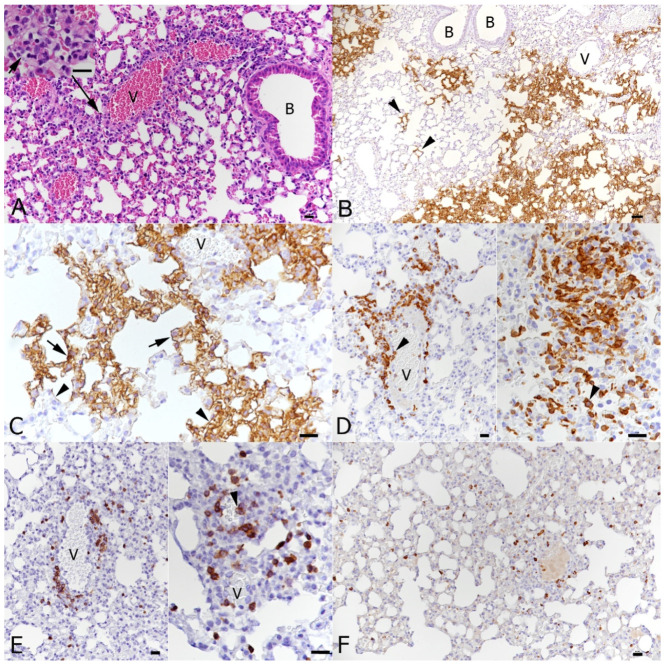
Lungs, K18-hACE2 transgenic mice, at day 3 post-infection with SARS-CoV-2. (**A**) There is mild perivascular mononuclear infiltration and the parenchyma exhibits mild multifocal activation of type II pneumocytes (inset). HE stain. (**B**,**C**) Staining for SARS-CoV-2 antigen reveals random multifocal areas of SARS-CoV-2 infection, affecting both individual alveoli ((**B**): arrowheads) and large parenchymal areas. Viral antigen expression is seen in type I pneumocytes ((**C**): arrowheads) and type II pneumocytes ((**C**): arrows). (**D**) Staining for macrophages (Iba-1+) shows recruitment from (left image, arrowhead: monocytes attached to the endothelium of a vein) and accumulation of monocytes around veins, macrophage accumulation in the parenchyma and desquamation of alveolar macrophages (right image, arrowhead). (**E**) T cells (CD3+) are less numerous than macrophages and are mainly found in the perivascular infiltrates. They are also recruited from the blood (right image, arrowhead). (**F**) B cells (CD45R/B220+) are seen in low numbers, disseminated in the parenchyma. Immunohistology, hematoxylin counterstain (**B**–**F**). B: bronchiole; V: vessel. Bars = 20 μm.

**Figure 6:**
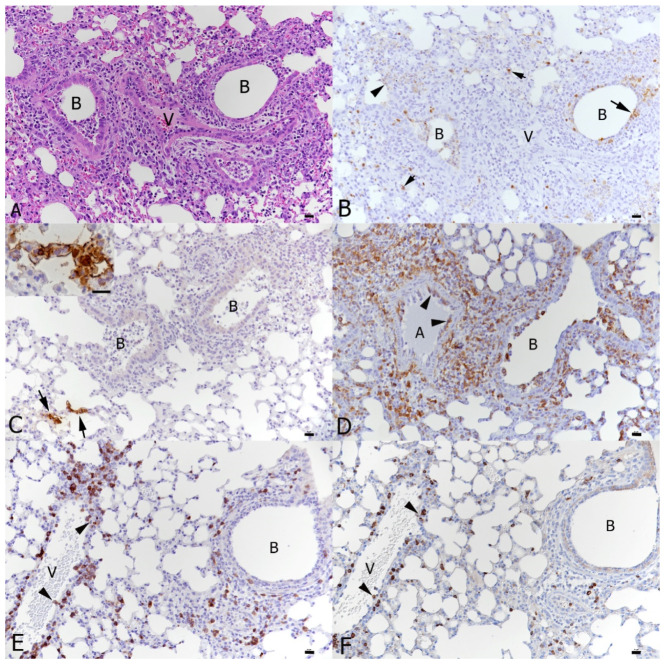
Lungs, K18-hACE2 transgenic mice, at day 6 post-infection with IAV and day 3 post-infection with SARS-CoV-2 in double infection. (**A**) The IAV-associated changes dominate (see also Figure 4E). HE stain. (**B**) This is confirmed by staining for IAV antigen which is detected in bronchiolar epithelial cells (arrow), occasional type I pneumocytes (arrowhead) and disseminated type II pneumocytes (short, small arrows). (**C**) SARS-CoV-2 infection is seen in areas not affected by IAV-induced changes ((**B**): bronchioles with IAV changes) and mainly in individual alveoli where both type I and type II pneumocytes are found to express viral antigen (inset). (**D**) Macrophages (Iba1+) are abundant around affected bronchioles and in the exudate in the bronchiolar lumen and are recruited from the blood into the perivascular infiltrates (arrowheads: rolling and emigrating monocytes). (**E**) T cells (CD3+) are recruited in moderate numbers from the blood (arrowheads) into the perivascular infiltrates. (**F**) B cells (CD45R/B220+) are recruited in low numbers from the blood (arrowheads) into the perivascular infiltrates. Immunohistology, hematoxylin counterstain (**B**–**F**). A: artery; B: bronchiole; V: vessel. Bars = 20 μm.

At 6 days post-IAV infection, the transgenic mice exhibited the pulmonary changes typically seen in wild-type mice after IAV X31 infection at this time point [40] We observed epithelial cell degeneration and necrosis in several bronchioles which also contained debris in the lumen as well as occasional small focal peribronchial areas where alveoli also exhibited necrotic cells (Figure 4B). The IAV antigen was found in epithelial cells in bronchi and bronchioles, in type I and II pneumocytes in affected alveoli and in a few randomly distributed individual type II pneumocytes [40]. Vessels showed evidence of lymphocyte recruitment, vasculitis and perivascular lymphocyte infiltration. A comparative assessment of the lungs in wild-type mice at the same time point post-infection confirmed that the genetic manipulation indeed had no effect on the response of mice to IAV infection [40]. At the comparative time point, a SARS-CoV-2 single infection (day 6, 3 dpi) was associated with mild changes, represented by a mild increase in interstitial cellularity, evidence of type II pneumocyte activation (Figure 4C and Figure 5A), occasional desquamated alveolar macrophages/type II pneumocytes and single erythrocytes in alveolar lumina and multifocal, predominantly perivascular mononuclear infiltration with the recruitment of leukocytes from vessels (Figure 4D). Infiltrating cells were predominantly macrophages, with T cells mainly around vessels and a low number of disseminated B cells (Figure 5); macrophages and T cells were also found to emigrate from veins (Figure 5D,E), as recently reported [40]. A viral antigen was found in multifocal patches of individual to large groups of alveoli, randomly distributed throughout the parenchyma, within type I and type II pneumocytes (Figure 5C), but not within bronchiolar epithelial cells (Figure 5B). Double infection at this time point, i.e., 6 days after IAV infection and 3 days after SARS-CoV-2 infection, was associated with histological changes almost identical to those induced by IAV, although they appeared to be slightly more extensive (Figure 4E,F). IAV antigen expression had a distribution and extent similar to that seen in a single IAV infection at the same time point. It was observed in epithelial cells in bronchi and bronchioles, in type I and II pneumocytes in affected alveoli and in few randomly distributed individual type II pneumocytes (Figure 6B). SARS-CoV-2 antigen expression was less intense than in SARS-CoV-2-only infected mice. This was observed in random individual or small groups of alveoli (Figure 6C) and in type I and II pneumocytes (Figure 6C inset).

**Figure 7:**
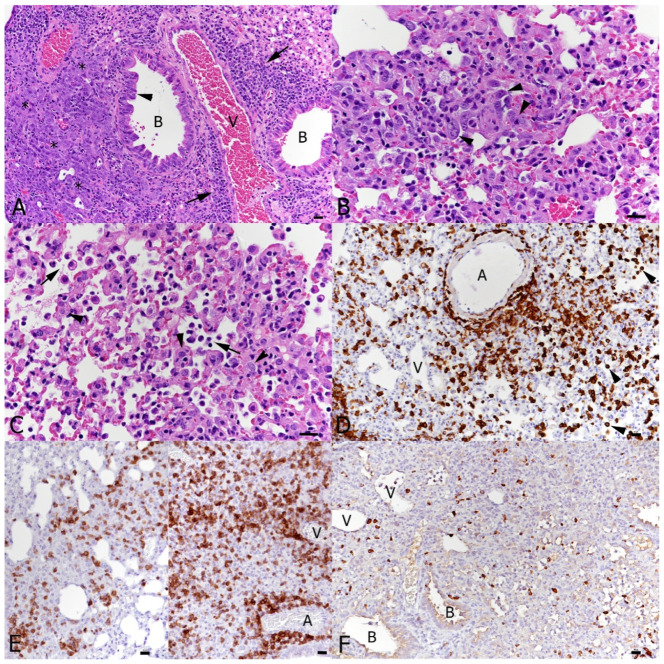
Lungs, K18-hACE2 transgenic mice, at day 10 post-infection with IAV and day 7 post-infection with SARS-CoV-2 in single infections. (**A**) IAV-infected animal; 10 dpi. Bronchioles (**B**) exhibit epithelial hyperplasia (arrowhead) and there is respiratory epithelial cell/type II pneumocyte hyperplasia (*) in the adjacent parenchyma. Vessels (V) exhibit variably intense lymphocyte-dominated perivascular infiltrates (arrows). (**B**–**F**). SARS-CoV-2-infected animal; 7 dpi. (**B**) There are abundant activated type II pneumocytes which also show syncytia formation (arrowheads). (**C**) There are also focal changes consistent with acute pneumonia, with desquamation of alveolar macrophages/type II pneumocytes (arrows) and type II pneumocyte activation (arrowheads). (**D**) Macrophages (Iba1+) are abundant in perivascular infiltrates and within the altered parenchyma. (**E**) T cells (CD3+) are also abundant in the parenchymal infiltrates. (**F**) B cells (CD45R/B220+) are seen in low numbers disseminated in the parenchymal infiltrates. A: artery; B: bronchiole; V: vessel. HE stain (**A**–**C**); immunohistology, hematoxylin counterstain (**D**–**F**). Bars = 20 μm.

Four days later, at the endpoint of the experiment, i.e., at 10 days after IAV infection and 7 days of SARS-CoV-2 infection, the histopathological features had changed. Single IAV infection had by then almost entirely resolved; however, the lungs exhibited changes consistent with a regenerative process, i.e., mild-to-moderate hyperplasia of the bronchiolar epithelium with adjacent multifocal respiratory epithelial cell/type II pneumocyte hyperplasia, together with mild-to-moderate lymphocyte-dominated perivascular infiltration (Figure 7A). At this stage, the effect of SARS-CoV-2 infection was more evident. A single infection had resulted in multifocal areas with distinct type II pneumocyte activation and syncytial cell formation (Figure 7B), mononuclear infiltration, leukocyte recruitment and perivascular infiltration. There were also a few focal areas of mild desquamative pneumonia with intra-alveolar macrophages/type II pneumocytes, edema and fibrin deposition (Figure 7C). Macrophages and T cells dominated in the infiltrates (Figure 7D,E), whereas B cells were found disseminated in low numbers (Figure 7F). 

**Figure 8:**
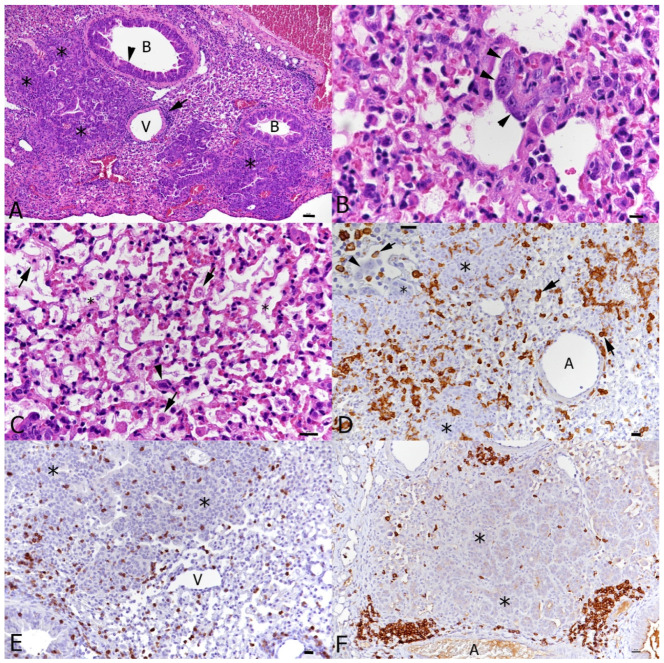
Lungs, K18-hACE2 transgenic mice, at day 10 post-infection with IAV and day 7 post-infection with SARS-CoV-2 in double infection. (**A**) There are abundant changes consistent with those seen in single IAV-infected mice, i.e., epithelial cell hyperplasia in bronchioles (**B**), multifocal type II pneumocyte hyperplasia (*) and perivascular lymphocyte-dominated infiltrates (arrow). (**B**,**C**) Changes attributable to SARS-CoV-2 infection. These comprise type II pneumocyte activation and syncytia formation ((**B**): arrowheads) and acute pneumonia (**C**), with desquamation of alveolar macrophages/type II pneumocytes (arrows) and type II pneumocyte activation (arrowheads). In more severe cases, alveoli occasionally contain fibrin and hyaline membranes (*). (**D**) Macrophages (Iba1+) form focal parenchymal infiltrates and are found around areas of type II pneumocyte hyperplasia (*). There are also desquamated alveolar macrophages (Iba1+; arrows). The lack of Iba1 expression in syncytial cells confirms that they are type II pneumocytes (inset: arrowhead). (**E**) T cells (CD3+) are present in moderate numbers throughout the infiltrates and around areas of type II pneumocyte hyperplasia (*). (**F**) B cells (CD45R/B220+) form occasional small aggregates in proximity to areas of type II pneumocyte hyperplasia (*). A: artery; B: bronchiole; V: vessel. HE stain (**A**–**C**); immunohistology, hematoxylin counterstain (**D**–**F**). Bars = 20 μm.

The SARS-CoV-2-associated changes were also observed in the double-infected mice (Figure 8C–F), where they were generally more pronounced (Figure 8B,C) and present alongside equally pronounced regenerative changes attributable to IAV infection (moderate hyperplasia of the bronchiolar epithelium with adjacent multifocal respiratory epithelial cell/type II pneumocyte hyperplasia; Figure 8A). Also, in this group of animals, macrophages were the dominant infiltrating cells. However, the number of T cells was comparatively low (Figure 8D,E). B cells were generally rare, but occasionally formed small aggregates in proximity to areas of epithelial hyperplasia (Figure 8F). Interestingly, the pattern of viral antigen expression had not changed with time; SARS-CoV-2 NP was detected in type I and II pneumocytes of unaltered-appearing alveoli.

**Figure 9:**
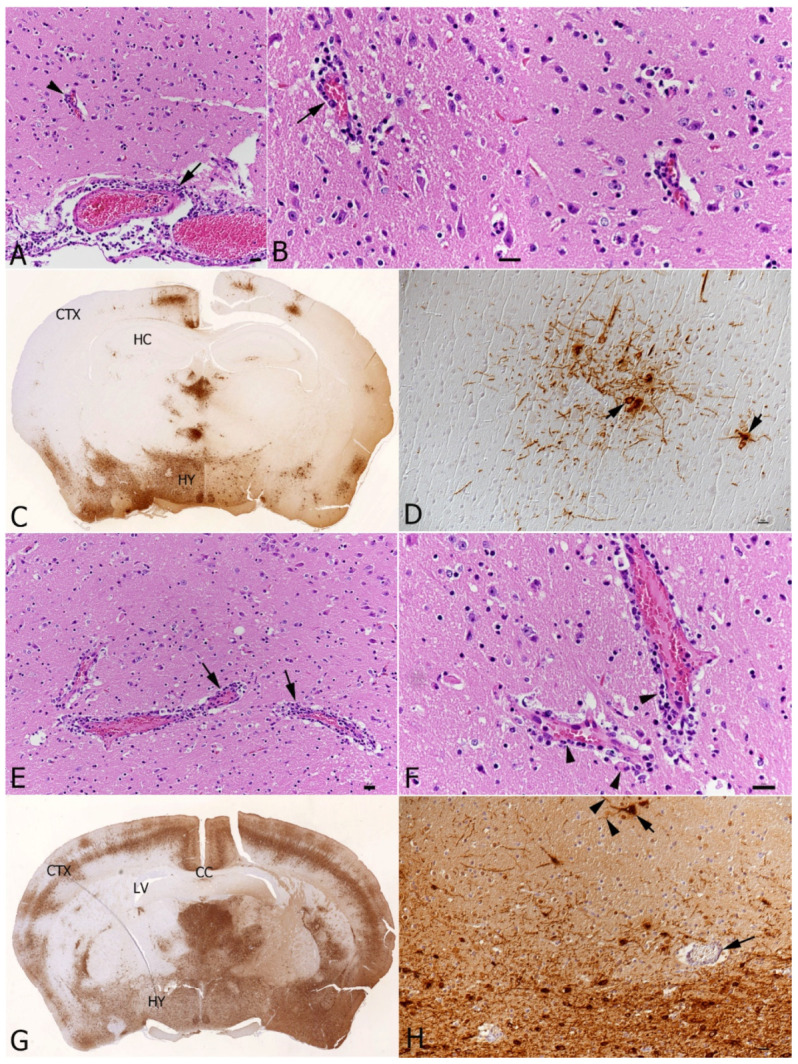
Brain, K18-hACE2 transgenic mice, at day 7 post-single-infection with SARS-CoV-2 or at day 10 post-infection with IAV and day 7 post-infection with SARS-CoV-2 in double infections. (**A**–**D**) SARS-CoV-2 single infection. (**A**,**B**) Hypothalamus. (**A**) Vessels in the leptomeninx (arrow) and in the brain parenchyma (arrowhead) exhibit mild perivascular mononuclear infiltrations, consistent with mild non-suppurative meningoencephalitis. (**B**) Higher magnification highlighting the one-layered perivascular infiltrate (arrows). There are a few degenerate cells (right image; arrowheads). (**C**) Coronal section at the level of the hippocampus (HC), showing extensive SARS-CoV-2 antigen expression in the hypothalamus (HY) and bilateral patchy areas with positive neurons also in the cortex (CTX). (**D**) A higher magnification of a focal area with SARS-CoV-2 expression shows that infection is in the neurons (arrowheads). (**E**–**H**) IAV and SARS-CoV-2 double-infected K18-hACE2 transgenic mouse. (**E**,**F**) The perivascular mononuclear infiltrate is slightly more intense than in the SARS-CoV-2 single-infected mouse ((**E**): arrows), consistent with moderate non-suppurative encephalitis. Among the perivascular infiltrate are several degenerate cells ((**F**): arrowheads). (**G**) Coronal section at the level of the corpus callosum (CC), showing extensive widespread bilateral SARS-CoV-2 antigen expression (HY: hypothalamus; CTX: cortex; LV: left ventricle). (**H**) Closer view showing viral antigen expression in neuronal cell bodies (short arrow) and processes (arrowheads). Long arrow: vessel with mild perivascular infiltration. HE stain (**A**,**B**,**E**,**F**); immunohistology, hematoxylin counterstain (**C**,**D**,**G**,**H**). Bars = 20 μm.

In two of the four single SARS-CoV-2-infected and three of the four double-infected mice at the later time point (7 days post-SARS-CoV-2 infection), we observed mild or moderate non-suppurative meningoencephalitis, mainly affecting the midbrain and brainstem (Figure 9). Changes were consistent with those previously reported for these mice [36] and appeared more severe in the double-infected mice, where the perivascular infiltrates contained degenerate leukocytes and appeared to be associated with the focal loss of the integrity of the endothelial cell layer (Figure 9B).

The effect of Fluenz Tetra immunisation on the lungs of the mice was assessed by histology and immunohistology. On day 6 after its application, the lungs exhibited a mild multifocal increase in interstitial cellularity, mild-to-moderate multifocal mononuclear (macrophages and lymphocytes) peribronchial and perivascular infiltration and evidence of mild leukocyte emigration. There were very rare IAV antigen positive intact individual bronchiolar epithelial cells and type II pneumocytes (Supplementary Figure S1A). SARS-CoV-2 coinfection (3 dpi) was associated with the same histological changes and IAV antigen expression, while SARS-CoV-2 antigen expression was not observed (Supplementary Figure S1B). An examination of the nasal mucosa at this stage found IAV antigen expression restricted to a few, partly degenerate respiratory epithelial cells in one animal (Supplementary Figure S1C, left), while SARS-CoV-2 antigen expression was found in individual and small patches of intact and occasionally degenerate respiratory epithelial cells (Supplementary Figure S3C, right). The olfactory epithelium and brain were negative. At day 10 post-Fluenz Tetra, histological changes in the lungs were restricted to a mildly increased interstitial cellularity and mild peribronchial and perivascular infiltration by lymphocytes and fewer plasma cells (Supplementary Figure S1D). At this stage, SARS-CoV-2 coinfection (7 dpi) did not result in further changes in two of the four animals; neither showed SARS-CoV-2 NP expression in the lung, but there was evidence of infection, represented by a few individual respiratory epithelial cells in the nasal mucosa that were positive for the viral antigen. The remaining two animals showed changes consistent with SARS-CoV-2 infection, i.e., focal consolidated areas with several macrophages, activated type II cells and occasional syncytial cells, some lymphocytes and neutrophils and a few degenerate cells (Supplementary Figure S1E). SARS-CoV-2 NP expression was seen in a few individual macrophages within the focal lesions and in rare alveoli (type I and II epithelial cells) in one animal and in several patches of alveoli in the second (Supplemental Figure S1F). In the first animal, infected epithelial cells were not found in the nasal mucosa, but there were individual neurons and a few larger patches of positive neurons and neuronal processes in the frontal cortex and brain stem. In the second animal, the brain was negative, but there were a few SARS-CoV-2 antigen positive respiratory epithelial cells in the nasal mucosa.

### 3.4. Distinct Transcriptional Signatures Are Associated with Infection

The transcriptional profile of lung samples can provide a window to view the host response to infection by a respiratory pathogen. Therefore, lung samples were taken at day 6 and day 10 post-IAV or -Fluenz Tetra infection from all groups of mice (Figure 1). Total RNA was purified from cells and both host and viral mRNA (and genomic RNA in the case of SARS-CoV-2) were sequenced on the NovaSeq illumina platform.

**Figure 10:**
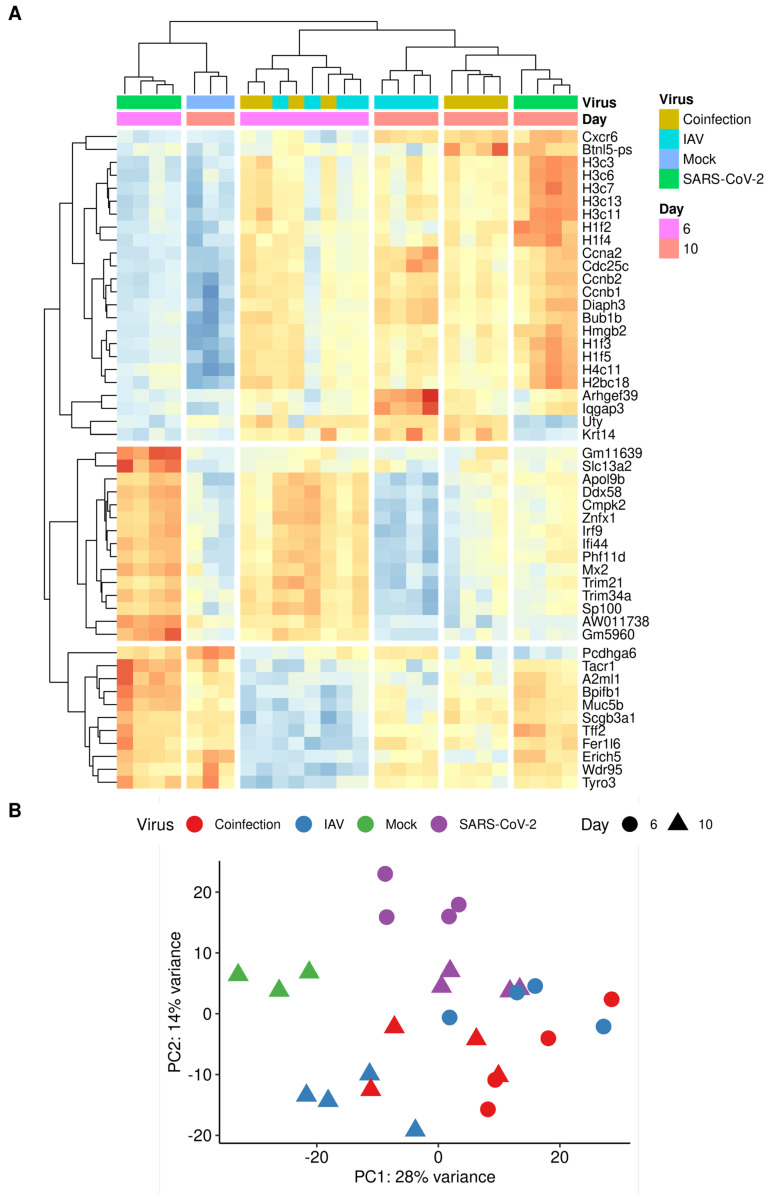
RNA sequencing analysis of lung homogenates from K18-hACE2 mice infected with either IAV only, SARS-CoV-2 only or IAV and SARS-CoV-2 (n = 3–5). (**A**) The top 50 differentially expressed gene transcripts across 4 groups at 2 time-points are shown. (**B**) Principal component analysis performed for 28 samples with log2 transformed counts per million (cpm).

**Figure 11:**
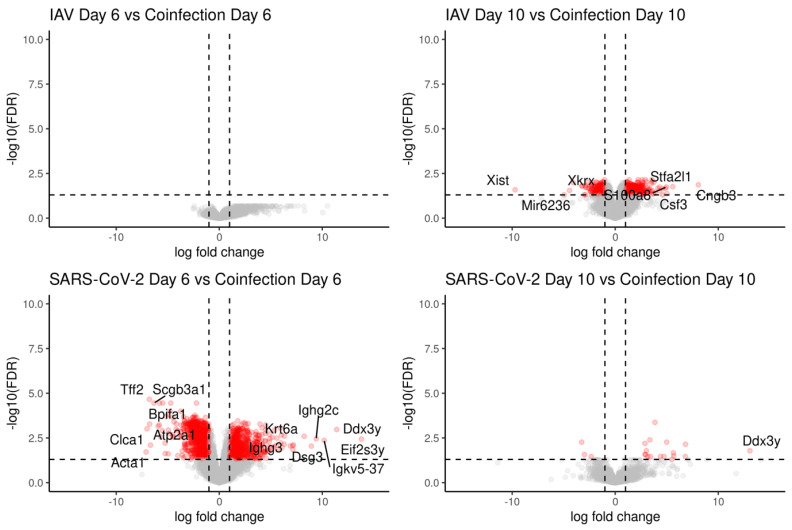
Coinfected mice at day 6 had a similar transcriptomic profile to IAV-only infected mice at day 6, whereas coinfected mice at day 10 were more similar to SARS-CoV-2-infected mice at day 10, with a few distinct differences. To identify transcripts that were different in each condition, contrasts were made between single infection and coinfection at day 6 and day 10 following a comparison to mock infection. The upregulated genes represent genes that have a higher abundance in the coinfected group in comparison to the singly infected group. The numbers of differentially expressed genes are presented in Table 2. There were no significantly changed transcripts when comparing the expression of transcripts in IAV only and coinfection at day 6, suggesting these groups were transcriptionally similar. Data points colored in red signify genes with an FDR < 0.05 and LogFC ± 2.

Transcripts were counted against the *Mus musculus* transcriptome using Salmon [41]. Gene expression was inferred with tximport and normalised using the edgeR package before identifying differentially expressed genes, using the transcription profile from mock-infected mice as the control profile. A total of 22,101 genes were identified and the number of transcripts significantly changing in abundance is presented in Table 2. The top 50 differentially expressed genes form clusters (through the pheatmap hclust parameter) based on the experimental groups, where at day 6, single-IAV and coinfection belong to the same cluster. By day 10, each experimental group can be distinguished by the transcript expression (Figure 10A). Principle component analysis (PCA) revealed separation between the IAV and SARS-CoV-2 groups and overlap between coinfection and IAV infection groups (Figure 10B). Overlapping signatures likely represent the non-specific anti-viral response. Contrast matrices were made between mice that were coinfected versus mice that were mock-infected and mice that were singly infected (Table 2). When comparing coinfected mice to IAV-only infection at day 6, there were no significant transcripts identified to have changed in abundance; however, by day 10, there were significant differences (Table 2; Figure 11). The coinfected mice had significant changes in comparison to SARS-CoV-2-only infected mice at both day 6 and day 10, where more differences were observed at day 6 (Table 2; Figure 11). 

**Figure 12:**
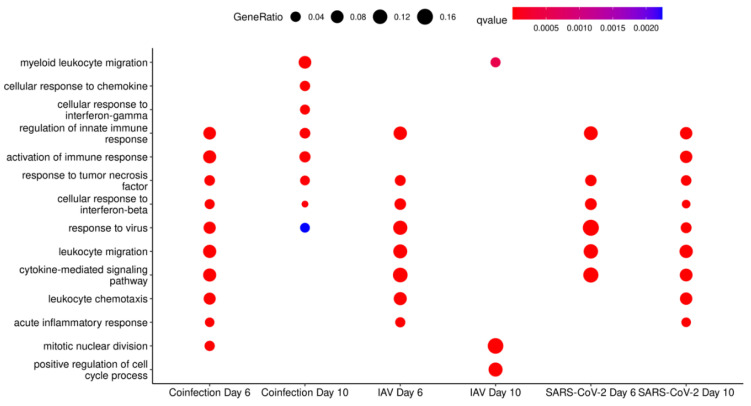
Selected Biological Process GO Terms derived from transcripts in increased abundance when comparing infection groups to mock-infected mice at day 6 and day 10 in clusterProfiler.

To assign ontology to the transcripts identified during the differential gene expression analysis, the clusterProfiler was used to provide the biological process, molecular function and cellular component gene ontology terms. Overall, the terms identified show an upregulation of innate immune responses (Figure 12). By day 10, the IAV group does not show an enrichment for these terms, indicative of infection; however, normal cellular processes such as “positive regulation of cell cycle” and “nuclear division” are observed, supporting that this experimental group has resolved the IAV infection and that regenerative processes were taking place [40]. Interestingly, SARS-CoV-2-only infected mice maintain terms representative of an active viral infection; however, they do not show enrichment in “leukocyte chemotaxis”, “chemokine production” and “cellular response to interferon gamma”, as seen in the coinfected group. Cellular components (Supplementary Figure S2) and molecular functions (Supplementary Figure S3) are also presented. Cytokine activity molecular functions are upregulated at day 6 and day 10 in coinfected mice and are otherwise only seen at day 6 in IAV-only infected mice.

**Figure 13:**
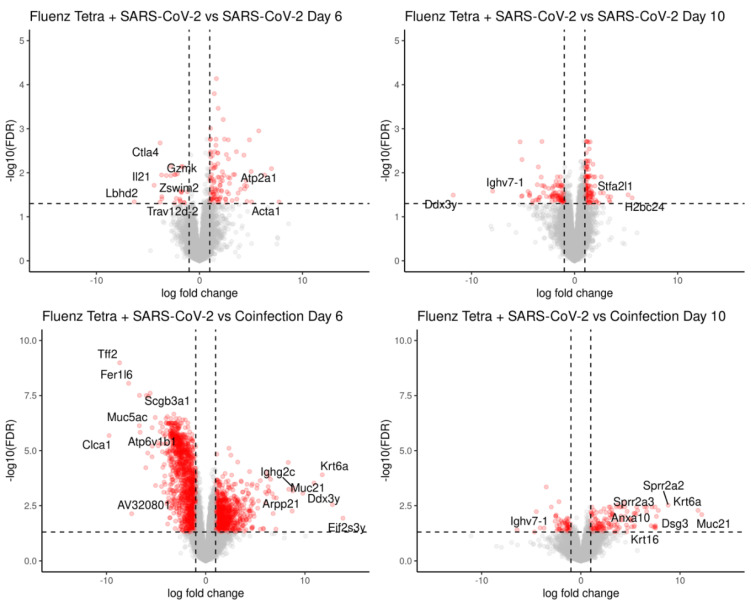
Key differences in transcript abundances between SARS-CoV-2-only infection and coinfection against SARS-CoV-2 infection with the addition of Fluenz Tetra were identified by comparing the two conditions after comparison to mock-infected mice. Genes identified as “up” represent a higher abundance in SARS-CoV-2-infected only or coinfection, whereas “down” represents a higher abundance in Fluenz Tetra and SARS-CoV-2. Data points coloured in red signify genes with an FDR < 0.05 and LogFC ± 2.

Comparing the SARS-CoV-2-only and SARS-CoV-2 infection following Fluenz Tetra immunisation, a total of 82 significant differentially expressed transcripts were identified at day 6 and a total of 71 at day 10 (Table 3). Interestingly, at day 6, interferon-stimulated genes (ISGs) such as Ifit1, Ifit3 and Trim69 were in higher abundance in SARS-CoV-2-infected mice (Supplementary Table S1, Figure 13). As SARS-CoV-2 singly infected mice exhibited respiratory signs and weight loss, it can be postulated that the regulation of these transcripts are important factors of disease severity. By day 10, transcripts associated with adaptive immune responses were found in higher abundance in the Fluenz Tetra-immunised SARS-CoV-2-infected mice (Igha, Ighg1, Ighv1–64, Ighv1–75, Ighv5–17, Ighv7–1, Jchain, Igkv13–84, Igkv3–5, Igkv10–96, Igkv12–44, Igkv14–111 and Igkv4–57-1) (Supplementary Table S2, Figure 13). The plethora of transcripts that are higher in abundance in the Fluenz Tetra-immunised mice may encode molecules that offer some protection; however, immunisation also dampens the expression of transcripts associated with interferon signalling.

**Figure 14:**
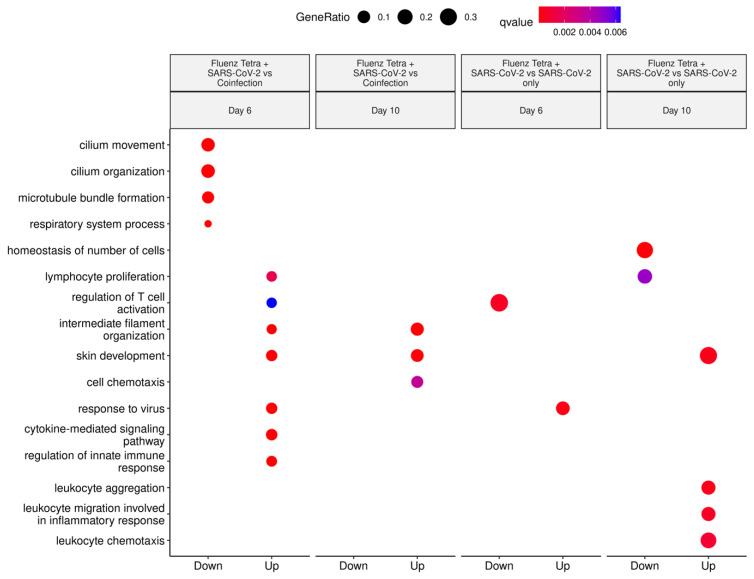
Selected Biological Process GO Terms derived from transcripts increasing and decreasing in abundance when comparing the Fluenz Tetra and SARS-CoV-2-infected group with SARS-CoV-2 only or coinfection groups (following comparison to mock-infected group). Clusters identified as “up” represent a higher abundance in SARS-CoV-2-infected only or coinfection, whereas “down” represents a higher abundance in Fluenz Tetra and SARS-CoV-2.

Transcripts that were found to be increasing and decreasing comparatively were inputted into the ClusterProfiler to assign gene ontology terms for the biological process, molecular function and cellular components. The biological process clusters demonstrate that the Fluenz Tetra-immunised and SARS-CoV-2-infected mice have transcripts associated with the “Regulation of T cell activation” in higher abundance than mice infected with SARS-CoV-2 only at day 6, whereas the “response to virus” was higher in the SARS-CoV-2-only infected group. By day 10, the SARS-CoV-2-infected group showed an increase in transcripts associated with “leukocyte aggregation”, the “leukocyte migration involved in inflammatory response” and “leukocyte chemotaxis”, further demonstrating that SARS-CoV-2-driven pathological processes are driven by host inflammation pathways (Figure 14).

When comparing SARS-CoV-2 infection following Fluenz Tetra immunisation to the coinfection group, 1259 differentially expressed genes were identified at day 6 and 140 at day 10 (Table 3). A gene ontology assessment with the clusterProfiler revealed enrichment in biological processes such as “cilium movement” and “cilium organization” in comparison to the coinfected mice. Many genes within this cluster belonged to the cilia- and flagella-associated protein (cfap) family and are downregulated during infection (Figure 13), as seen previously in the IAV-only infection [40]. Additionally, processes such as the “cytokine-mediated signaling pathway” and the “regulation of innate immune response” are upregulated in coinfected animals in comparison to the vaccine group, demonstrating that Fluenz Tetra immunisation prevents transcriptional activity, indicative of pulmonary damage. Cellular components (Supplementary Figure S4) and molecular functions (Supplementary Figure S5) are also presented.

## 4. Discussion

In this study, the sequential intranasal infection of K18-hACE2 mice with IAV followed by SARS-CoV-2 led to a more severe pulmonary disease than infections with IAV or SARS-CoV-2 alone. Following IAV infection, mice coinfected with SARS-CoV-2 displayed significantly higher weight loss, elevated respiratory distress and more rapid mortality compared to mice infected with IAV alone. A transcriptomics analysis revealed that the expression of several genes specific to airway epithelial cells were more downregulated in coinfected mice compared to singly infected mice at days 6 and 10, which is indicative of increased lung epithelial cell damage. This is possibly driven by the IAV infection, where previously we have seen significant ‘epithelial alterations’ associated with cilia [40]. Interestingly, coinfected mice exhibited significantly lower levels of SARS-CoV-2 viral RNA and sgRNA at day 6 (3 dpi with SARS-CoV-2) compared to SARS-CoV-2 singly infected mice, indicating that whilst coinfection results in enhanced respiratory signs, existing IAV infection interferes with the infection of SARS-CoV-2 at this time point. Furthermore, pre-vaccination with an attenuated quadrivalent influenza vaccine (Fluenz Tetra) also elicited a reduction in SARS-CoV-2 viral RNA and sgRNA; however, this was in the absence of the advanced weight loss and clinical signs observed in the SARS-CoV-2–IAV-coinfected mice. These findings were recapitulated by the analysis and comparison of the pathological changes in the lungs. Overt pulmonary damage was due to IAV and was represented by multifocal epithelial cell necrosis in the bronchioles and adjacent alveoli. SARS-CoV-2 infection was also multifocal but restricted to alveoli distant from those affected by IAV, consistent with infection via aerosol from the upper respiratory tract and reflecting that both viruses compete for their target cells in the alveoli; accordingly, the destruction of the alveoli by IAV could explain the lower SARS-CoV-2 loads in double-infected mice. By day 10, coinfected mice and SARS-CoV-2 singly infected mice displayed similar levels of viral RNA and sgRNA, suggesting that whilst initially inhibited by the presence of IAV, SARS-CoV-2 was able to overcome this inhibition and achieve unconstrained replication. In the Fluenz Tetra-vaccinated mice challenged with SARS-CoV-2, viral RNA and sgRNA copies remained undetectable in 2/4 animals, indicating that the immune response triggered by the attenuated influenza virus is restrictive to SARS-CoV-2 infection. This was reflected in the lung transcriptome profile that showed a sustained innate response in coinfected animals over the time period of both infections. A histological examination indicated that at 10 days post-infection, the damage induced by IAV was resolving. There were distinct regenerative changes, represented by bronchiolar epithelial cell (and type II pneumocyte) hyperplasia, accompanied by a moderate macrophage-dominated inflammatory response. SARS-CoV-2 infection still showed the same distribution pattern, with viral antigen expression in epithelial cells of unaltered-appearing alveoli, but not in new epithelial cells in areas of hyperplasia, confirming that, like other coronaviruses, SARS-CoV-2 also only infects fully differentiated epithelial cells. However, there was evidence of SARS-CoV-2-induced damage, represented by the syncytia formation of type II pneumocytes and more pronounced alveolar damage (acute desquamative pneumonia and occasional hyaline membrane formation). With the SARS-CoV-2 single infection, the inflammatory response appeared to be less macrophage-dominated as T cells were present in similar amounts. The two Fluenz Tetra-vaccinated mice in which SARS-CoV-2 viral RNA and sgRNA was detected also exhibited SARS-CoV-2-associated histological changes and viral antigen expression, though to a lesser degree than the double-infected animals, further confirming the limiting effect of influenza virus immunisation on SARS-CoV-2 infection and damage in the lung. 

Viral Interference is a well-documented phenomenon which has previously been reported between influenza B viruses (IBV) in a ferret model, in which infection with one IBV subtype was able to prevent infection with another subtype when infections were separated by 3 days [42]. Similar to the observations described herein between IAV and SARS-CoV-2, the coinfection of antigenically unrelated viruses such as IAV and IBV did not confer resistance when challenged within 3 days, but merely delayed the shedding of the challenge virus. This delay in shedding likely accounts for the differing times at which coinfected animals and SARS-CoV-2 singly infected animals lost weight. SARS-CoV-2 singly infected animals exhibited weight loss at 4 dpi; however, coinfected animals began to recover from IAV infection before succumbing to the delayed SARS-CoV-2 infection. Unlike this study, wherein IAV and SARS-CoV-2 coinfected animals exhibited significantly increased weight loss, coinfection with IAV and IBV has been reported to lead to delayed viral shedding, but did not influence disease severity [28].

Mathematical modelling and in vitro and in vivo studies have shown that prior infection with rhinovirus interferes with IAV infection [6]. This interference is mediated by the induction of interferon-stimulated genes (ISGs) following rhinovirus infection which work to suppress IAV. Similarly, infection with IAV results in the activation of the IFN response and the upregulation of ISGs which induce an antiviral state that works to limit infection [43] (and reviewed [44]). We propose that this response is also active in the K-18-hACE2 mice 3 days post-IAV infection and contributes to the inhibition of the incoming SARS-CoV-2 infection, thus resulting in a lower viral load as measured by RT-qPCR at day 6. The IAV viral load was found to be similar between coinfected and IAV singly infected mice, demonstrating that SARS-CoV-2 infection does not interfere with prior IAV infection. Similarly, by day 10 (7 days post-SARS-CoV-2 infection), both coinfected and IAV singly infected mice were negative for IAV by immunohistology and qPCR, indicating that SARS-CoV-2 infection does not prolong IAV infection or interfere with the ability of the immune system to clear IAV infection. At this stage, the IAV singly infected mice exhibited histological features consistent with regeneration, including hyperplasia of the bronchiolar epithelium and type II pneumocytes. Conversely, while the coinfected mice also displayed evidence of epithelial regeneration, they also presented several hallmarks of acute lung injury including alveolar edema, fibrin exudation, hyaline membrane formation and the degeneration and desquamation of alveolar epithelial cells. This elevated lung injury is consistent with the SARS-CoV-2 loads present in these animals at day 10. 

Compared to mice coinfected with IAV and SARS-CoV-2, Fluenz Tetra-immunised SARS-CoV-2-infected mice displayed reduced ISG expression at 6 dpi, but an elevated expression of transcripts associated with T cell and lymphocyte regulation. Interestingly, an increase in immunoglobulin transcripts, T cell activation and adaptive immune responses has been shown to be associated with patients who went on to survive from COVID-19 in a point-of-care study [45], further reinforcing aberrant immune responses as drivers of disease severity. The reduction in ISG transcripts in the Fluenz Tetra-immunised mice may be due to the low IAV load found in these mice, owing to the attenuation of the virus strains included in the vaccination. Fluenz Tetra does not contain an adjuvant; therefore, the observed stimulation of innate and adaptive immune response transcripts is due to viral infection alone (European Medicines Agency, https://www.ema.europa.eu/en/medicines/human/EPAR/fluenz-tetra (accessed on 1 March 2024)). Since Fluenz Tetra was able to reduce SARS-CoV-2 viral loads, but without the increase in disease severity associated with IAV infection, it can be postulated that the innate immune response elicited by the pathogenic IAV infection contributes to the enhanced disease severity displayed by coinfected animals. As 2/4 Fluenz Tetra-vaccinated mice displayed no detectable SARS-CoV-2 viral RNA at days 6 and 10 and no evidence of NP staining in the lungs, this preimmunisation appears to have rendered them refractory to SARS-CoV-2 infection. 

Interestingly, a similar coinfection study which utilised the IAV strain A/WSN/33 (WSN) found similar pathological findings to this study following coinfection; however, they reported that prior infection with IAV enhanced SARS-CoV-2 infection [46]. IAV infection in vitro was found to enhance ACE2 expression, thereby promoting SARS-CoV-2 infection, potentially due to the modulation of ACE2 expression by IFN [46,47]. This enhancement was also noted in vivo using the K18-hACE2 mouse model; however, the expression of hACE2 in this model was not upregulated following IAV infection. The observed enhancement of SARS-CoV-2 infection, therefore, cannot be attributed to the IFN response elicited by WSN infection and the authors postulate that the observed enhancement of SARS-CoV-2 pathogenicity is a unique feature of IAV infection. The observed differences between these results and the results presented herein may be explained by the doses of the virus used and the timings of the infections. The study in which IAV enhanced SARS-CoV-2 infection utilised a higher dose of IAV (2000 PFU) compared to the dose used in our study (100 PFU) and the mice were infected with SARS-CoV-2 a day earlier, at 2 dpi compared to 3 dpi. As IAV is capable of antagonising the IFN response early in infections, this increased dose may result in more potent reduction of the IFN response. Similarly, the early IFN response may be reduced at 2 dpi compared to 3 dpi, thereby promoting SARS-CoV-2 infection. Unfortunately, the authors did not quantify the IFN response in the coinfected mice, nor carry out transcriptomic analysis, so direct comparisons are not possible. Future studies utilising mouse-adapted SARS-CoV-2 [48,49] are needed to explore the relationship between SARS-CoV-2 and IAV coinfection in more detail, as the ACE2 expression in these mice will be stimulated more naturally following IAV infection instead of relying on the K18 promoter.

As reported in studies using K18-hACE2 transgenic mice [36,50,51], some of the SARS-CoV-2 singly infected and coinfected mice had developed non-suppurative meningoencephalitis by day 10, predominantly affecting the midbrain and brainstem. As shown an in-depth study of our group on SARS-CoV-2 associated encephalitis [36], the virus spreads to the brain after the initial bout of respiratory infection, usually from day 4 onwards. The distribution of the virus antigen and inflammatory changes are consistent with the ascending infection from the nasal cavity via the olfactory bulb [36,52]. Interestingly, the coinfected mice displayed a more substantial virus spread in the brain and more pronounced perivascular infiltration with evidence of structural blood–brain barrier (BBB) breakdown. The mechanism through which coinfection with IAV may enhance SARS-CoV-2 neurological infection is unclear. While brain infection has been well documented in cases of influenza [53,54,55], this is predominantly limited to neurotropic and highly pathogenic strains and occurs via the breakdown of the BBB following high levels of viremia [54,56]. BBB integrity is also reduced by proinflammatory cytokines such as IL-6, IL-1β and IFN-γ which disrupt the tight junctions maintained by brain microvascular endothelial cells (reviewed in [57]). While the IAV X31 strain used herein did not result in brain infection, it is possible that the increased cytokine response present in coinfected animals further compromised the BBB integrity and allowed increased leukocyte recruitment. As brain invasion was only apparent in 1/4 of the Fluenz Tetra-immunised mice post-SARS-CoV-2 infection, it can be postulated that the innate immune response raised by attenuated IAV was sufficient to reduce SARS-CoV-2 infectivity, without resulting in a pathogenic immune response associated with increased neuroinvasion. The increased pathogenicity associated with coinfection can, therefore, be the consequence of the pathological overstimulation of the innate immune response.

No animal model can predict with absolute certainty the consequences of coinfection in humans. However, the data presented here may have critical implications for the development of successful pre-emptive interventions for SARS-CoV-2. Fortunately, public health interventions aimed at delaying the transmission of SARS-CoV-2 should also provide a consequent reduction in the transmission of the influenza virus if they are effectively implemented. Moreover, some but not all experimental therapeutics being studied for SARS-CoV-2 have also been demonstrated to exhibit activity against the influenza virus. As for other viruses for which successful antiviral interventions have been developed, the SARS-CoV-2 polymerase has emerged as a strong initial target for small-molecule inhibitors. Importantly, drugs such as remdesivir and favipiravir that are in various stages of development and clinical evaluation for SARS-CoV-2 have direct or metabolite-driven in vitro activity against the influenza virus [58,59], with favipiravir also approved for influenza in Japan. Other examples of dual-activity against these viruses are evident with other small-molecule antivirals such as nitazoxanide [60,61,62] and niclosamide [63,64], which may present opportunities and/or a basis for the prioritisation of candidates for clinical evaluation if necessary exposures can be achieved [65,66]. Such antiviral interventions have potential application in the treatment of early infection as well as the prophylactic setting. Chemoprevention is a particularly attractive approach when we move into winter months and the selection of the right candidates for evaluation may provide a benefit for both viruses individually and in coinfections. It should be noted that many of the advanced technologies (e.g., broadly neutralising monoclonal antibodies) that are being rapidly accelerated through development have explicit specificities that provide high potency, but this is likely to preclude activity against viruses other than those against which they are directed. The work presented here shows that an experimental setting would be an effective pre-clinical platform with which to test therapeutic approaches to dealing with coinfection which is pertinent with the return of seasonal flu outbreaks concomitant with reoccurring global outbreaks of SARS-CoV-2 VOCs.

## 5. Conclusions

The present study shows that the sequential infection of IAV and SARS-CoV-2 is associated with a more severe disease phenotype, supported by histological and transcriptomics analysis. Prior infection with IAV or Fluenz Tetra reduces SARS-CoV-2 RNA synthesis, where the infection with Fluenz Tetra is not associated with an enhanced disease phenotype and an aberrant immune response. This reinforces the need for public health strategies that consider the potential impact of the coinfection of circulating respiratory viruses. 

## Figures and Tables

**Table 1:** Study groups and treatments given to the different individual groups of mice.

Group	Day 0	Day 3
Control	PBS	PBS
IAV	IAV	PBS
SARS-CoV-2	PBS	SARS-CoV-2
IAV + SARS-CoV-2	IAV	SARS-CoV-2
Fluenz Tetra	Fluenz Tetra	PBS
Fluenz Tetra + SARS-CoV-2	Fluenz Tetra	SARS-CoV-2

**Table 2:** Number of differentially expressed genes with an FDR value less than 0.05 and a log2 fold change more than 2 or less than −2 compared to mock-infected mice (row 1) and coinfected mice (rows 2 and 3). Coinfection day 6 and day 10 were compared to day 6 and 10 of individual IAV and SARS-CoV-2 infection.

Group	Up/ Down	IAV Alone		SARS-CoV-2 Alone		Coinfection	
		D6	D10	D6	D10	D6	D10
Mock D10	Up	628	357	335	777	973	548
	Down	547	121	53	369	707	184
Coinfection D6	Up	0	-	327	-	-	-
	Down	0	-	480	-	-	-
Coinfection D10	Up	-	150	-	20	-	-
	Down	-	59	-	3	-	-

Abbreviations: D—day.

**Table 3:** Number of transcripts changing in abundance and an FDR ≤ 0.05 when comparing SARS-CoV-2-only infection and coinfection to SARS-CoV-2 infection following Fluenz Tetra immunisation at day 6 and day 10.

Group	Up/Down	SARS-CoV-2		Coinfection	
		D6 vs. Mock	D10 vs. Mock	D6 vs. Mock	D10 vs. Mock
FT + SARS-CoV-2 D6 vs. Mock	Up	57	-	416	-
	Down	25	-	843	-
FT + SARS-CoV-2 D6 vs. Mock	Up	-	17	-	108
	Down	-	54	-	32

Abbreviations: D—day; FT—Fluenz Tetra.

## Data Availability

Code used to analyse RNA sequencing data is available at https://github.com/Hiscox-lab/k18-hACE2-coinfection-transcriptomics (accessed on 1 March 2024). Sequencing readings are available under BioProject ID: PRJNA914664 in Short Read Archive (SRA).

## Supplementary Materials

The following supporting information can be downloaded at: https://www.mdpi.com/article/10.3390/v16060863/s1, Figure S1: Histological changes and viral antigen expression in Fluenz Tetra-immunised mice; Figure S2: Coinfection cellular component enrichment analysis; Figure S3: Coinfection molecular function enrichment analysis; Figure S4: Fluenz Tetra cellular component enrichment analysis; Figure S5: Fluenz Tetra molecular function enrichment analysis; Table S1: Differentially expressed genes; Fluenz Tetra and SARS-CoV-2 infection vs. SARS-CoV-2-only infection at day 6; Table S2: Differentially expressed genes; Fluenz Tetra and SARS-CoV-2 infection vs. SARS-CoV-2-only infection at day 10.
