# Supplementary material for: Sequential Infection with Influenza A Virus Followed by Severe Acute Respiratory Syndrome Coronavirus 2 (SARS-CoV-2) Leads to More Severe Disease and Encephalitis in a Mouse Model of COVID-19
Source: Viruses. 2024 May 28;16(6):863. doi: 10.3390/v16060863 (PMC11209060; doi:10.3390/v16060863)
Supplement: Supplementary file 1 [file viruses-16-00863-s001.zip › viruses-2902112-supplementary.pdf]

Supplementary Material

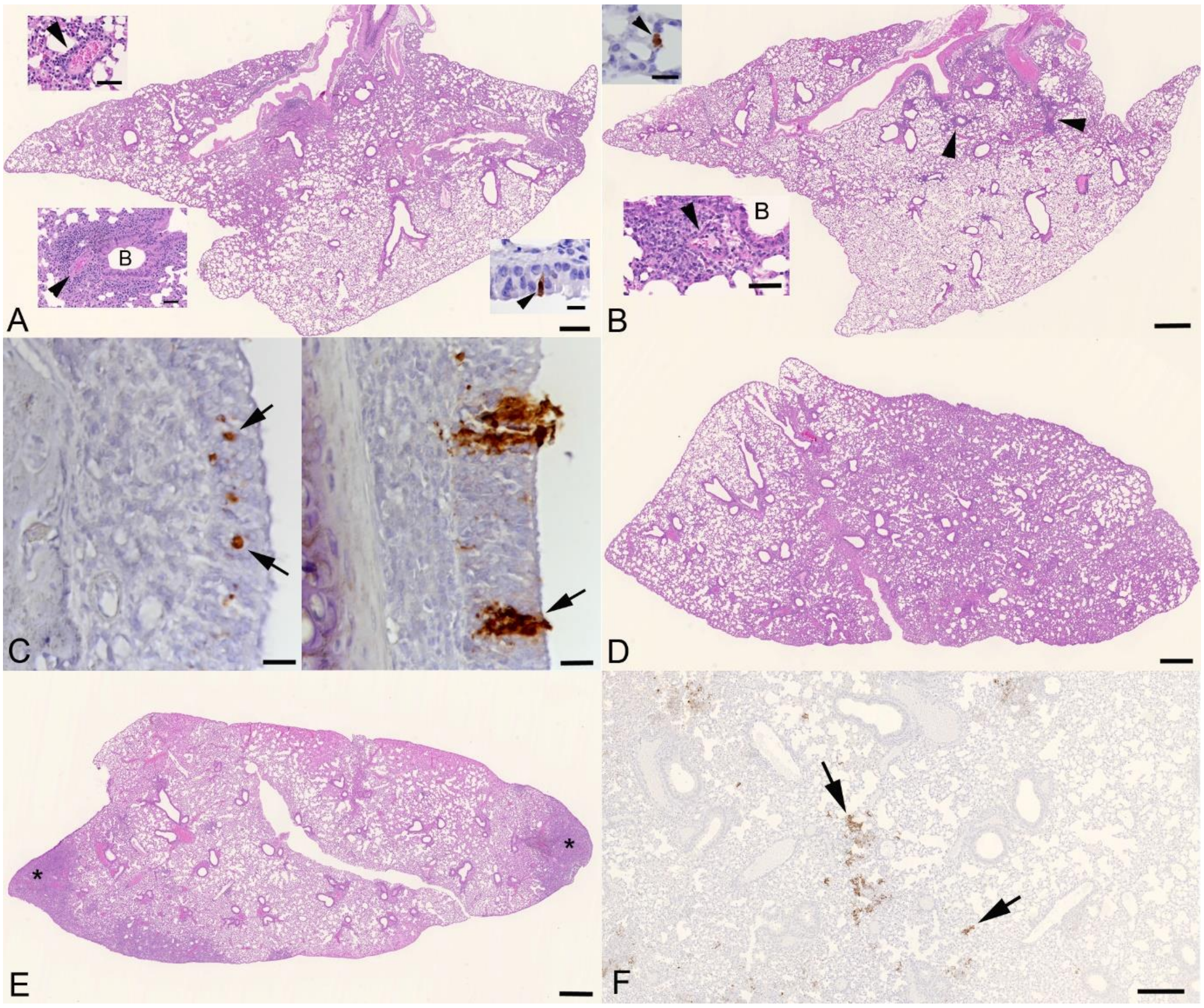

Figure S1. Histological changes and viral antigen expression in Fluenz Tetra immunized mice. **A)** Fluenz Tetra immunized mouse, 6 days post inoculation. Overview of left lung and insets showing mild perivascular (inset top left: arrowhead) and peribronchial (inset bottom left: arrowhead) mononuclear infiltration. IAV antigen expression is seen in rare individual bronchiolar epithelial cells (right inset: arrowhead). Bars = 500 µm (overview) and 50 µm (insets). **B, C)** Fluenz Tetra immunized mouse with SARS-CoV-2 co-infection at 3 days post inoculation, examined at 6 days post inoculation with Fluenz Tetra. **B)** Overview of left lung with focal perivascular infiltrates (arrowheads) as well as focal leukocyte rolling and emigration from vessels (bottom inset: arrowhead). IAV antigen expression is seen in scattered pneumocytes (top inset: arrowhead). Bar = 500 µm (overview), 50 µm (bottom inset) and 20 µm (top inset). **C)** Nasal mucosa. IAV antigen in a few respiratory epithelial cells (left), SARS-CoV-2 NP expression in patches of respiratory epithelial cells (right). Bars = 20 µm. **D)** Fluenz Tetra immunized mouse, 10 days post inoculation. Overview of the widely unaltered left lung. Bar = 500 µm. **E, F)** Fluenz Tetra immunized mouse with SARS-CoV-2 co-infection at 3 days post inoculation, examined at 10 days post inoculation with Fluenz Tetra. **E)** Overview of the left lung, showing focal consolidated areas (*) with infiltrating leukocytes, some activated type II pneumocytes and a few degenerate cells. Bar = 500 µm. **F)** SARS-CoV-2 NP expression is seen in a few patches of

alveoli. Bar = 250 μm HE stain (A, B, D, E); immunohistology, hematoxylin counterstain (insets A, B; C, F).

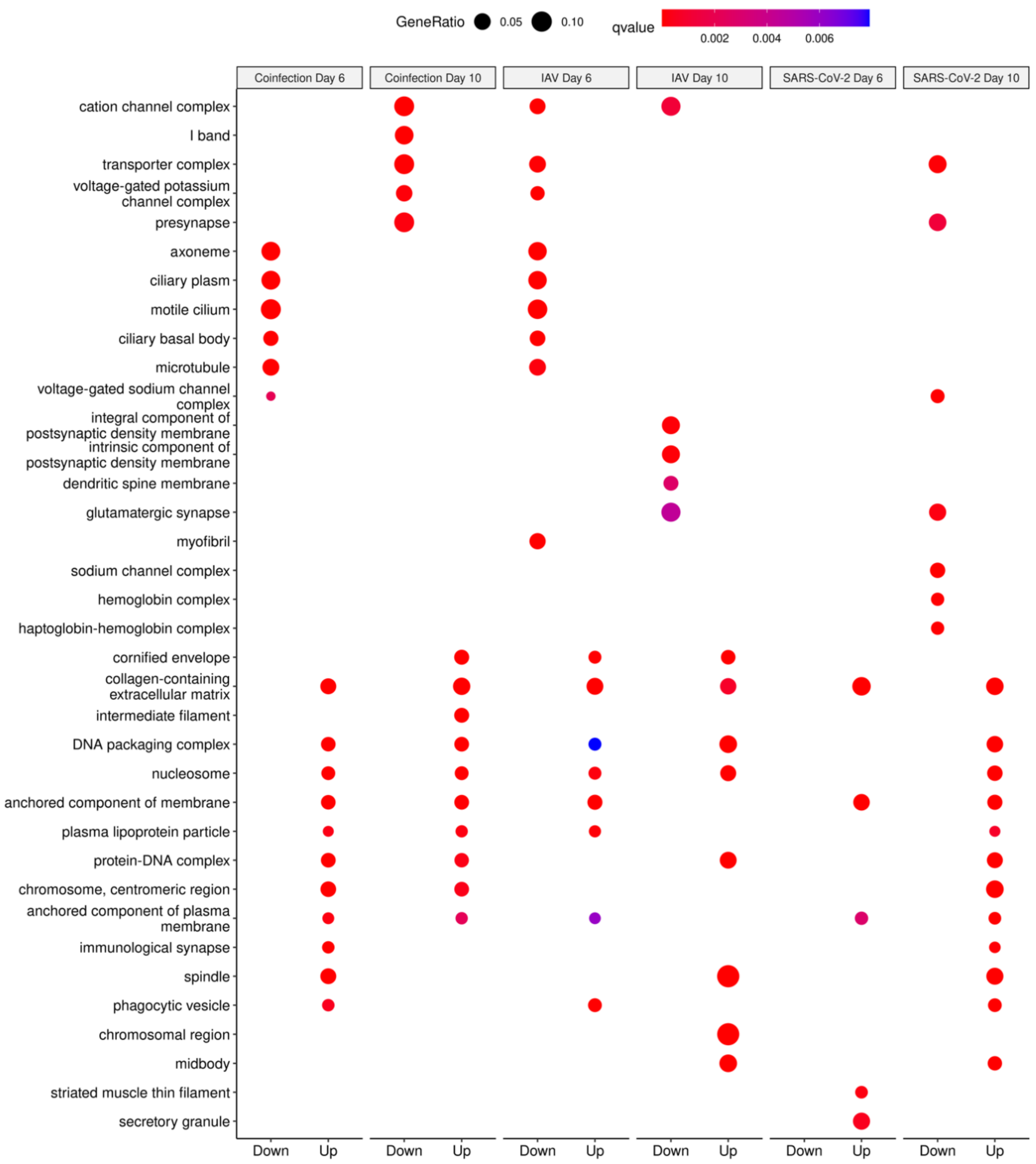

Figure S2. **Coinfection cellular component enrichment analysis**. The top 10 cellular component terms reported from clusterProfiler to assess gene enrichment following differential gene expression analysis.

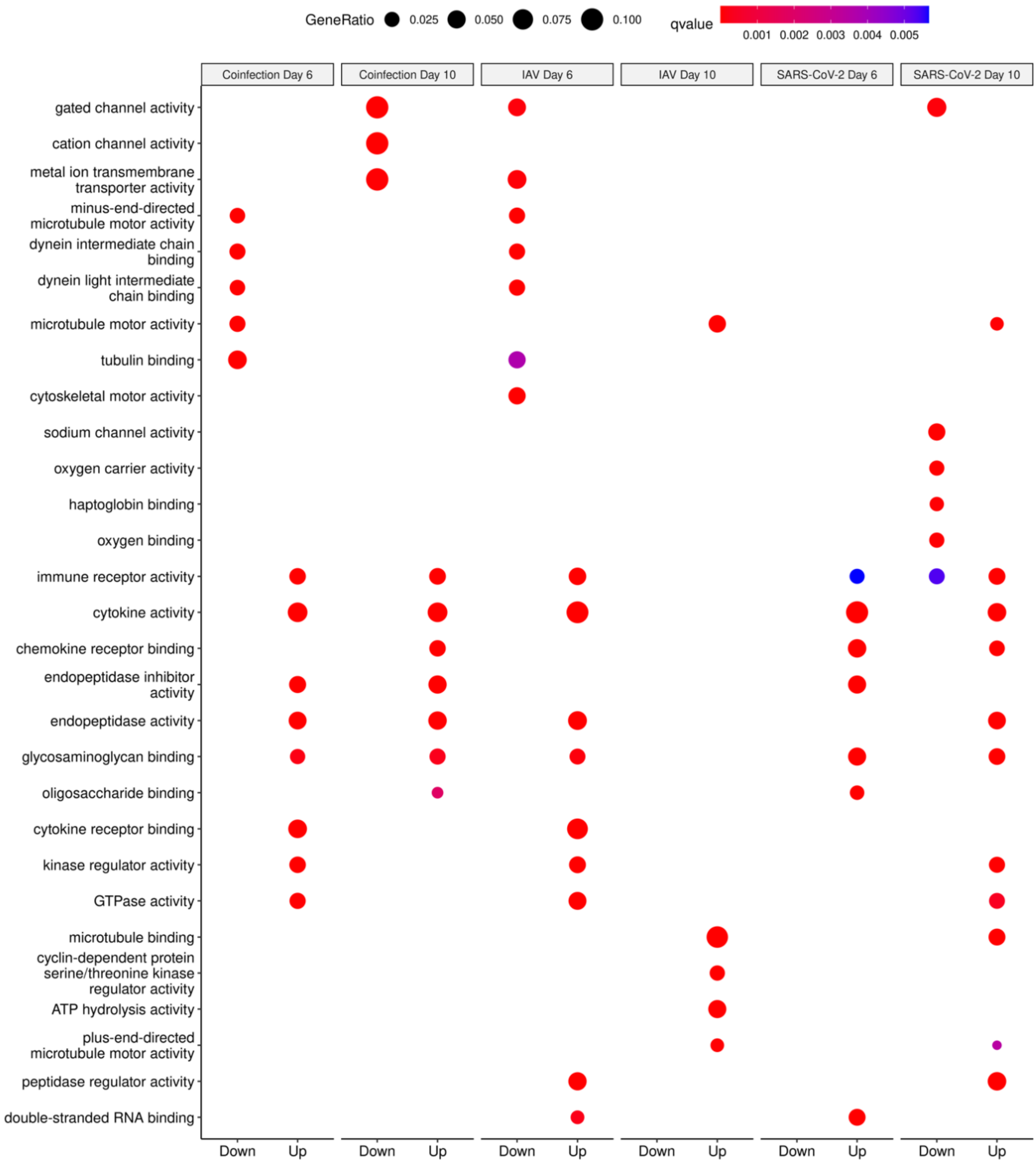

Figure S3. **Coinfection molecular function enrichment analysis**. The top 10 molecular function terms reported from clusterProfiler to assess gene enrichment following differential gene expression analysis.

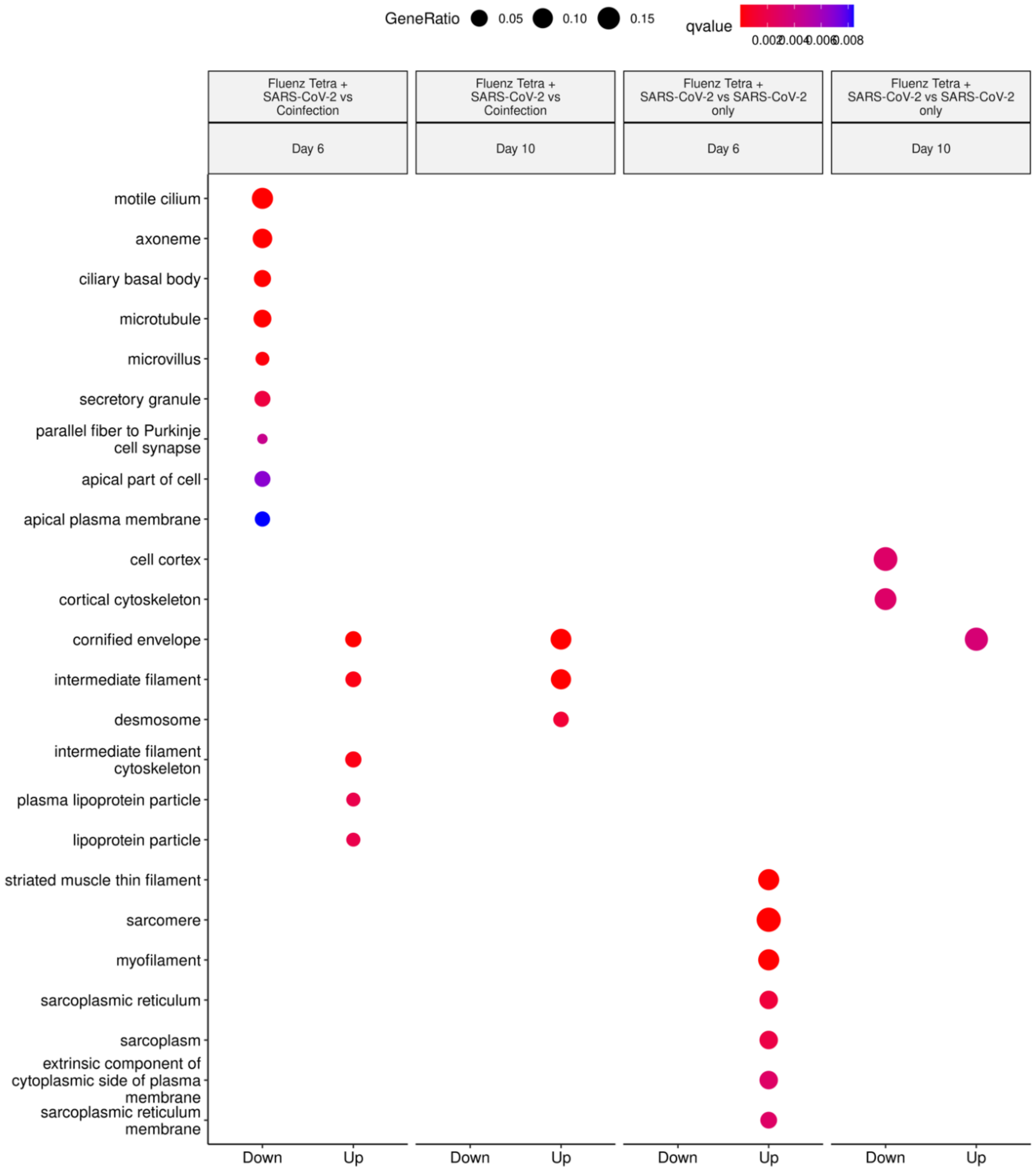

Figure S4. **Fluenz Tetra cellular component enrichment analysis.** Cellular component GO Terms derived from transcripts increasing and decreasing in abundance when comparing the Fluenz Tetra and SARS-CoV-2 infected group and the SARS-CoV-2 only infected group (following comparison to mock infected mice). Clusters identified in “up” represent a higher abundance in the SARS-CoV-2 only infected group, whereas “down” represents a higher abundance in the Fluenz Tetra and SARS-CoV-2 infected group.

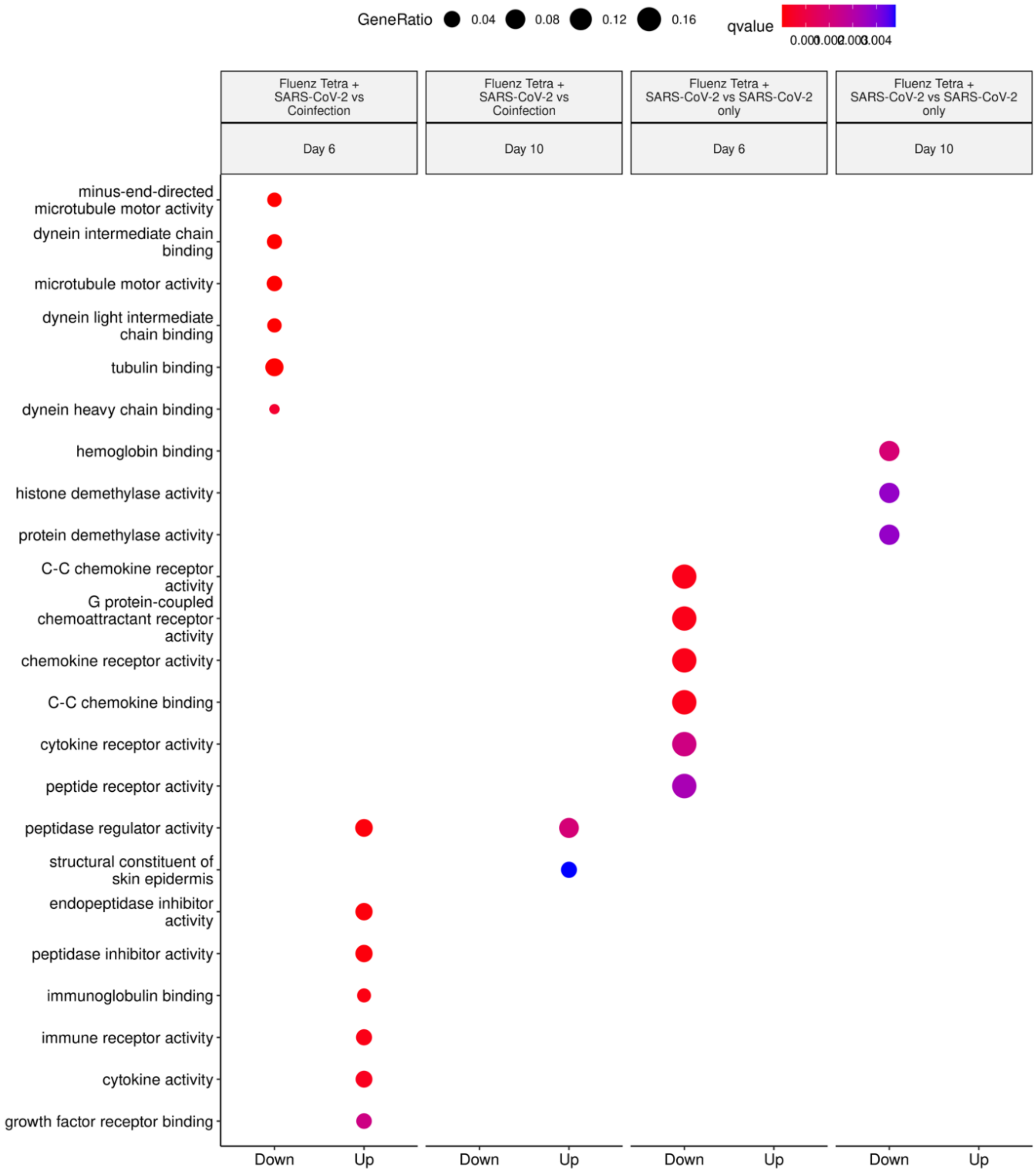

Figure S5. **Fluenz Tetra molecular function enrichment analysis.** Molecular function GO terms derived from transcripts increasing and decreasing in abundance when comparing the Fluenz Tetra and SARS-CoV-2 infected group and the SARS-CoV-2 only infected group (following comparison to mock infected mice). Clusters identified in “up” represent a higher abundance in the SARS-CoV-2 only infected group, whereas “down” represents a higher abundance in the Fluenz Tetra and SARS-CoV-2 infected group.

Supplementary Table S1. Differentially expressed genes; Fluenz Tetra and SARS-CoV-2 infection vs SARS-CoV-2 only infection at day 6.

Gene name	logFC	FDR	Description
ENSMUSG00002074901	9.94	4.613432e-02	predicted gene, 54819 [Source:MGI Symbol;Acc:MGI:6846115]
Gm12338	9.67	3.371104e-10	predicted gene 12338 [Source:MGI Symbol;Acc:MGI:3650622]
Acta1	7.72	4.614200e-02	actin alpha 1, skeletal muscle [Source:MGI Symbol;Acc:MGI:87902]
Atp2a1	6.99	8.069474e-03	ATPase, Ca++ transporting, cardiac muscle, fast twitch 1 [Source:MGI Symbol;Acc:MGI:105058]
Gm11639	6.40	3.146542e-06	predicted gene 11639 [Source:MGI Symbol;Acc:MGI:3651790]
Actn3	6.33	1.126126e-02	actinin alpha 3 [Source:MGI Symbol;Acc:MGI:99678]
Ryr1	5.75	1.120904e-03	ryanodine receptor 1, skeletal muscle [Source:MGI Symbol;Acc:MGI:99659]
Mrip-ps	5.07	4.611397e-02	Mom radiation induced polyposis, pseudogene [Source:MGI Symbol;Acc:MGI:3645947]
Tnnt3	5.02	9.374349e-03	troponin T3, skeletal, fast [Source:MGI Symbol;Acc:MGI:109550]
Apod	4.85	1.791848e-03	apolipoprotein D [Source:MGI Symbol;Acc:MGI:88056]
Mylpf	4.62	4.499558e-02	myosin light chain, phosphorylatable, fast skeletal muscle [Source:MGI Symbol;Acc:MGI:97273]
Casq1	4.59	2.018322e-02	calsequestrin 1 [Source:MGI Symbol;Acc:MGI:1309468]
Tnni2	4.54	1.523505e-02	troponin I, skeletal, fast 2 [Source:MGI Symbol;Acc:MGI:105070]
Xkrx	4.41	2.329359e-02	X-linked Kx blood group related, X-linked [Source:MGI Symbol;Acc:MGI:3584011]
Mylk2	4.37	1.941014e-02	myosin, light polypeptide kinase 2, skeletal muscle [Source:MGI Symbol;Acc:MGI:2139434]
Tmprss9	4.31	3.989206e-03	transmembrane protease, serine 9 [Source:MGI Symbol;Acc:MGI:3612246]
4930438A08Rik	4.19	1.904499e-02	RIKEN cDNA 4930438A08 gene [Source:MGI Symbol;Acc:MGI:1921238]
Fosl1	4.00	2.372947e-02	fos-like antigen 1 [Source:MGI Symbol;Acc:MGI:107179]
Slfn4	3.75	2.894643e-02	schlafen 4 [Source:MGI Symbol;Acc:MGI:1329010]
Neb	3.63	3.974895e-02	nebulin [Source:MGI Symbol;Acc:MGI:97292]
Gm5960	3.63	1.428918e-02	predicted gene 5960 [Source:MGI Symbol;Acc:MGI:3779540]
Coch	3.59	3.324672e-03	cochlin [Source:MGI Symbol;Acc:MGI:1278313]
A630012P03Rik	3.58	2.776423e-02	RIKEN cDNA A630012P03 gene [Source:MGI Symbol;Acc:MGI:2442968]
Mybpc2	3.43	1.098438e-02	myosin binding protein C, fast-type [Source:MGI Symbol;Acc:MGI:1336170]
Gm50419	3.43	3.684118e-03	
Rsad2	3.29	1.098438e-02	radical S-adenosyl methionine domain containing 2 [Source:MGI Symbol;Acc:MGI:1929628]
Gm31621	3.24	3.316564e-03	
Dusp13	3.17	4.280256e-02	dual specificity phosphatase 13 [Source:MGI Symbol;Acc:MGI:1351599]
Oasl1	2.96	1.326514e-02	2'-5' oligoadenylate synthetase-like 1 [Source:MGI Symbol;Acc:MGI:2180849]
Gm6034	2.96	1.752865e-02	predicted gene 6034 [Source:MGI Symbol;Acc:MGI:3646212]
Ifit3b	2.93	1.752865e-02	interferon-induced protein with tetratricopeptide repeats 3B [Source:MGI Symbol;Acc:MGI:3698419]

Dlgap1	2.83	3.020267e-02	DLG associated protein 1 [Source:MGI Symbol;Acc:MGI:1346065]
Gm47242	2.83	1.402876e-02	predicted gene, 47242 [Source:MGI Symbol;Acc:MGI:6096064]
Heatr9	2.81	4.846950e-03	HEAT repeat containing 9 [Source:MGI Symbol;Acc:MGI:3650286]
Lsmem1	2.81	1.267520e-02	leucine-rich single-pass membrane protein 1 [Source:MGI Symbol;Acc:MGI:2685735]
Mx2	2.80	1.791848e-03	
Mx1	2.68	2.706643e-02	
Trim69	2.61	3.858303e-03	tripartite motif-containing 69 [Source:MGI Symbol;Acc:MGI:1918178]
Ifit3	2.59	4.803425e-02	interferon-induced protein with tetratricopeptide repeats 3 [Source:MGI Symbol;Acc:MGI:1101055]
Ifit1	2.55	3.999282e-02	interferon-induced protein with tetratricopeptide repeats 1 [Source:MGI Symbol;Acc:MGI:99450]
Serpinb1c	2.52	3.944227e-02	serine (or cysteine) peptidase inhibitor, clade B, member 1c [Source:MGI Symbol;Acc:MGI:2445363]
Gm12551	2.50	4.611397e-02	predicted gene 12551 [Source:MGI Symbol;Acc:MGI:3651664]
Fap	2.43	1.733971e-03	fibroblast activation protein [Source:MGI Symbol;Acc:MGI:109608]
Npc1l1	2.39	1.143386e-02	NPC1 like intracellular cholesterol transporter 1 [Source:MGI Symbol;Acc:MGI:2685089]
A930015D03Rik	2.34	3.561906e-03	
Gm45418	2.33	3.143133e-02	predicted gene 45418 [Source:MGI Symbol;Acc:MGI:5791254]
Plekha4	2.31	6.200908e-04	pleckstrin homology domain containing, family A (phosphoinositide binding specific) member 4 [Source:MGI Symbol;Acc:MGI:1916467]
Gm6545	2.31	4.354765e-02	predicted gene 6545 [Source:MGI Symbol;Acc:MGI:3643874]
Mmp3	2.22	1.523505e-02	matrix metallopeptidase 3 [Source:MGI Symbol;Acc:MGI:97010]
Cmpk2	2.22	9.488447e-03	cytidine monophosphate (UMP-CMP) kinase 2, mitochondrial [Source:MGI Symbol;Acc:MGI:99830]
Usp18	2.16	1.267520e-02	ubiquitin specific peptidase 18 [Source:MGI Symbol;Acc:MGI:1344364]
Ifi44	2.15	2.659131e-02	interferon-induced protein 44 [Source:MGI Symbol;Acc:MGI:2443016]
Kcnq2	2.14	2.513158e-02	potassium voltage-gated channel, subfamily Q, member 2 [Source:MGI Symbol;Acc:MGI:1309503]
4930599N23Rik	2.10	2.792909e-02	
Perm1	2.05	1.609858e-02	PPARGC1 and ESRR induced regulator, muscle 1 [Source:MGI Symbol;Acc:MGI:1921433]
Dhx58	2.05	3.549059e-02	DEXH (Asp-Glu-X-His) box polypeptide 58 [Source:MGI Symbol;Acc:MGI:1931560]
Ptk6	2.02	1.941014e-02	PTK6 protein tyrosine kinase 6 [Source:MGI Symbol;Acc:MGI:99683]
4932415D10Rik	-2.06	4.611397e-02	RIKEN cDNA 4932415D10 gene [Source:MGI Symbol;Acc:MGI:3045298]
4933431J24Rik	-2.06	3.974895e-02	RIKEN cDNA 4933431J24 gene [Source:MGI Symbol;Acc:MGI:1918548]
Cxcr6	-2.13	1.065767e-02	chemokine (C-X-C motif) receptor 6 [Source:MGI Symbol;Acc:MGI:1934582]
Foxp3	-2.24	3.834554e-02	forkhead box P3 [Source:MGI Symbol;Acc:MGI:1891436]
Gm49146	-2.26	4.640065e-02	

Vmn2r96	-2.33	1.105706e-02	vomeronasal 2, receptor 96 [Source:MGI Symbol;Acc:MGI:3644514]
Hrh4	-2.42	4.859561e-02	histamine receptor H4 [Source:MGI Symbol;Acc:MGI:2429635]
Cxcr3	-2.44	1.098438e-02	chemokine (C-X-C motif) receptor 3 [Source:MGI Symbol;Acc:MGI:1277207]
Gm37039	-2.47	2.372947e-02	predicted gene, 37039 [Source:MGI Symbol;Acc:MGI:5610267]
Gm47345	-2.72	2.372947e-02	predicted gene, 47345 [Source:MGI Symbol;Acc:MGI:6096241]
Icos	-2.78	1.159781e-02	inducible T cell co-stimulator [Source:MGI Symbol;Acc:MGI:1858745]
Fer1l6	-2.78	7.183938e-03	fer-1-like 6 (C. elegans) [Source:MGI Symbol;Acc:MGI:3645398]
Gm39323	-2.82	1.904499e-02	predicted gene, 39323 [Source:MGI Symbol;Acc:MGI:5622208]
Gm10688	-3.12	2.770639e-02	predicted gene 10688 [Source:MGI Symbol;Acc:MGI:3642688]
Platr7	-3.21	1.159781e-02	
Gm48018	-3.59	2.967926e-03	
Ocm	-3.64	3.892387e-02	oncomodulin [Source:MGI Symbol;Acc:MGI:97401]
Zswim2	-3.65	3.487272e-02	zinc finger SWIM-type containing 2 [Source:MGI Symbol;Acc:MGI:1919111]
Gzmk	-3.67	1.126126e-02	granzyme K [Source:MGI Symbol;Acc:MGI:1298232]
Ctla4	-3.82	2.105702e-03	cytotoxic T-lymphocyte-associated protein 4 [Source:MGI Symbol;Acc:MGI:88556]
Trav12d-2	-3.83	4.656003e-02	T cell receptor alpha variable 12D-2 [Source:MGI Symbol;Acc:MGI:3642520]
Gm17266	-4.27	9.212213e-03	predicted gene, 17266 [Source:MGI Symbol;Acc:MGI:4936900]
Il21	-4.40	1.931471e-02	interleukin 21 [Source:MGI Symbol;Acc:MGI:1890474]
Gm35037	-6.24	2.513158e-02	predicted gene, 35037 [Source:MGI Symbol;Acc:MGI:5594196]
Lbhd2	-6.33	4.565424e-02	LBH domain containing 2 [Source:MGI Symbol;Acc:MGI:2685744]

Supplementary Table S2. Differentially expressed genes; Fluenz Tetra and SARS-CoV-2 infection vs SARS-CoV-2 only infection at day 10.

Gene name	logFC	FDR	Description
Gm14288	11.05	0.042590535	predicted gene 14288 [Source:MGI Symbol;Acc:MGI:3706570]
H2bc24	5.59	0.037078862	H2B clustered histone 24 [Source:MGI Symbol;Acc:MGI:3710645]
Stfa2l1	5.19	0.031694134	stefin A2 like 1 [Source:MGI Symbol;Acc:MGI:3524944]
S100a9	3.51	0.035215766	S100 calcium binding protein A9 (calgranulin B) [Source:MGI Symbol;Acc:MGI:1338947]
Xkrx	3.45	0.045911394	X-linked Kx blood group related, X-linked [Source:MGI Symbol;Acc:MGI:3584011]
S100a8	3.37	0.041855279	S100 calcium binding protein A8 (calgranulin A) [Source:MGI Symbol;Acc:MGI:88244]
Retnlg	3.21	0.034590256	resistin like gamma [Source:MGI Symbol;Acc:MGI:2667763]
Gm13282	2.83	0.031362230	predicted gene 13282 [Source:MGI Symbol;Acc:MGI:3649258]
Pbp2	2.80	0.028791539	phosphatidylethanolamine binding protein 2 [Source:MGI Symbol;Acc:MGI:1923650]
Topaz1	2.59	0.012436090	testis and ovary specific PAZ domain containing 1 [Source:MGI Symbol;Acc:MGI:3779933]
H2ac18	2.50	0.028791539	H2A clustered histone 18 [Source:MGI Symbol;Acc:MGI:96097]
Wnt10a	2.37	0.049055401	wingless-type MMTV integration site family, member 10A [Source:MGI Symbol;Acc:MGI:108071]
Gm8752	2.31	0.046891625	predicted pseudogene 8752 [Source:MGI Symbol;Acc:MGI:3647539]
Gm8797	2.14	0.048242754	predicted pseudogene 8797 [Source:MGI Symbol;Acc:MGI:3643769]
Asprv1	2.14	0.045416295	aspartic peptidase, retroviral-like 1 [Source:MGI Symbol;Acc:MGI:1915105]
H2ac4	2.10	0.046309739	H2A clustered histone 4 [Source:MGI Symbol;Acc:MGI:2448306]
Cldn4	2.05	0.041867826	claudin 4 [Source:MGI Symbol;Acc:MGI:1313314]
Gm43058	-2.02	0.028791539	predicted gene 43058 [Source:MGI Symbol;Acc:MGI:5663195]
5033403H07Rik	-2.02	0.032229611	
Sspo	-2.03	0.028771113	SCO-spondin [Source:MGI Symbol;Acc:MGI:2674311]
Hc	-2.06	0.020453051	hemolytic complement [Source:MGI Symbol;Acc:MGI:96031]
Gm26788	-2.09	0.043760343	predicted gene, 26788 [Source:MGI Symbol;Acc:MGI:5477282]
Gm3364	-2.10	0.019840700	predicted gene 3364 [Source:MGI Symbol;Acc:MGI:3781542]
Sox11	-2.10	0.030614777	SRY (sex determining region Y)-box 11 [Source:MGI Symbol;Acc:MGI:98359]
Alms1-ps1	-2.13	0.034590256	ALMS1, centrosome and basal body associated, pseudogene 1 [Source:MGI Symbol;Acc:MGI:3646394]
Catsperb	-2.15	0.034874748	cation channel sperm associated auxiliary subunit beta [Source:MGI Symbol;Acc:MGI:2443988]
Alas2	-2.16	0.022629305	aminolevulinic acid synthase 2, erythroid [Source:MGI Symbol;Acc:MGI:87990]
Hba-a2	-2.17	0.035789858	hemoglobin alpha, adult chain 2 [Source:MGI Symbol;Acc:MGI:96016]
Hbb-bt	-2.18	0.033197117	hemoglobin, beta adult t chain [Source:MGI Symbol;Acc:MGI:5474850]
Ndufs5-ps	-2.20	0.045100728	NADH:ubiquinone oxidoreductase core subunit S5, pseudogene [Source:MGI Symbol;Acc:MGI:3612445]
Gm38069	-2.21	0.047269311	predicted gene, 38069 [Source:MGI Symbol;Acc:MGI:5611297]
Snca	-2.25	0.033197117	synuclein, alpha [Source:MGI Symbol;Acc:MGI:1277151]
Gm43371	-2.26	0.032219268	predicted gene 43371 [Source:MGI Symbol;Acc:MGI:5663508]
Gm49674	-2.26	0.041274753	predicted gene, 49674 [Source:MGI Symbol;Acc:MGI:6215119]

Gm5737	-2.30	0.047349600	predicted gene 5737 [Source:MGI Symbol;Acc:MGI:3645276]
A630036G19Rik	-2.33	0.028523208	RIKEN cDNA A630036G19 gene [Source:MGI Symbol;Acc:MGI:2442979]
Aplnr	-2.40	0.035699347	apelin receptor [Source:MGI Symbol;Acc:MGI:1346086]
Gm48565	-2.43	0.038288185	predicted gene, 48565 [Source:MGI Symbol;Acc:MGI:6098124]
Gm43914	-2.45	0.012436090	predicted gene, 43914 [Source:MGI Symbol;Acc:MGI:5690306]
Epb42	-2.51	0.022629305	erythrocyte membrane protein band 4.2 [Source:MGI Symbol;Acc:MGI:95402]
9530059O14Rik	-2.63	0.006269374	RIKEN cDNA 9530059O14 gene [Source:MGI Symbol;Acc:MGI:2442421]
Gm39094	-2.66	0.022684974	predicted gene, 39094 [Source:MGI Symbol;Acc:MGI:5621979]
Svet1	-2.73	0.034681995	subventricular expressed transcript 1 [Source:MGI Symbol;Acc:MGI:2385655]
Olfr554	-2.86	0.021071989	olfactory receptor 554 [Source:MGI Symbol;Acc:MGI:3030388]
Prrt2	-2.99	0.019611443	proline-rich transmembrane protein 2 [Source:MGI Symbol;Acc:MGI:1916267]
Uty	-3.02	0.028366865	ubiquitously transcribed tetratricopeptide repeat containing, Y-linked [Source:MGI Symbol;Acc:MGI:894810]
Spta1	-3.17	0.001954992	spectrin alpha, erythrocytic 1 [Source:MGI Symbol;Acc:MGI:98385]
Gypa	-3.18	0.019611443	glycophorin A [Source:MGI Symbol;Acc:MGI:95880]
Hepacam2	-3.24	0.012577720	HEPACAM family member 2 [Source:MGI Symbol;Acc:MGI:2141520]
Igkv3-5	-3.36	0.035699347	immunoglobulin kappa chain variable 3-5 [Source:MGI Symbol;Acc:MGI:1330854]
Igkv4-57-1	-3.43	0.036540056	immunoglobulin kappa variable 4-57-1 [Source:MGI Symbol;Acc:MGI:2686264]
Gm47461	-3.47	0.041979673	predicted gene, 47461 [Source:MGI Symbol;Acc:MGI:6096425]
Ighv5-17	-3.48	0.041845801	immunoglobulin heavy variable 5-17 [Source:MGI Symbol;Acc:MGI:4439533]
Gm33677	-3.49	0.038076358	predicted gene, 33677 [Source:MGI Symbol;Acc:MGI:5592836]
Slc4a1	-3.52	0.007383358	solute carrier family 4 (anion exchanger), member 1 [Source:MGI Symbol;Acc:MGI:109393]
Btf3-ps1	-3.60	0.041049944	basic transcription factor 3, pseudogene 1 [Source:MGI Symbol;Acc:MGI:3781708]
Cd209a	-3.62	0.018715938	CD209a antigen [Source:MGI Symbol;Acc:MGI:2157942]
Lbhd2	-3.78	0.041846338	LBH domain containing 2 [Source:MGI Symbol;Acc:MGI:2685744]
Ighv1-64	-4.07	0.043396320	immunoglobulin heavy variable 1-64 [Source:MGI Symbol;Acc:MGI:4439789]
Ighv1-75	-4.08	0.049190936	immunoglobulin heavy variable 1-75 [Source:MGI Symbol;Acc:MGI:4439735]
Jchain	-4.35	0.033307414	immunoglobulin joining chain [Source:MGI Symbol;Acc:MGI:96493]
Kdm5d	-4.35	0.029425693	lysine (K)-specific demethylase 5D [Source:MGI Symbol;Acc:MGI:99780]
Igkv12-44	-4.41	0.032432726	immunoglobulin kappa variable 12-44 [Source:MGI Symbol;Acc:MGI:4439775]
Igkv10-96	-4.51	0.026588413	immunoglobulin kappa variable 10-96 [Source:MGI Symbol;Acc:MGI:4439561]
Igkv13-84	-5.09	0.033332225	immunoglobulin kappa chain variable 13-84 [Source:MGI Symbol;Acc:MGI:96514]
Igkv14-111	-5.10	0.005030811	immunoglobulin kappa variable 14-111 [Source:MGI Symbol;Acc:MGI:4439863]
Ighg1	-5.15	0.034590256	immunoglobulin heavy constant gamma 1 (G1m marker) [Source:MGI Symbol;Acc:MGI:96446]
Igha	-5.29	0.001989595	immunoglobulin heavy constant alpha [Source:MGI Symbol;Acc:MGI:96444]

U5	-7.03	0.049291158	predicted gene, 25313 [Source:MGI Symbol;Acc:MGI:5455090]
Ighv7-1	-7.95	0.026180276	immunoglobulin heavy variable 7-1 [Source:MGI Symbol;Acc:MGI:4439622]
Ddx3y	-11.78	0.032229611	DEAD box helicase 3, Y-linked [Source:MGI Symbol;Acc:MGI:1349406]
